# Spatio-temporal modeling of the crowding conditions and metabolic variability in microbial communities

**DOI:** 10.1371/journal.pcbi.1009140

**Published:** 2021-07-22

**Authors:** Liliana Angeles-Martinez, Vassily Hatzimanikatis

**Affiliations:** Laboratory of Computational Systems Biotechnology, École Polytechnique Fédérale de Lausanne, EPFL, Lausanne, Switzerland; University of Cambridge, UNITED KINGDOM

## Abstract

The metabolic capabilities of the species and the local environment shape the microbial interactions in a community either through the exchange of metabolic products or the competition for the resources. Cells are often arranged in close proximity to each other, creating a crowded environment that unevenly reduce the diffusion of nutrients. Herein, we investigated how the crowding conditions and metabolic variability among cells shape the dynamics of microbial communities. For this, we developed CROMICS, a spatio-temporal framework that combines techniques such as individual-based modeling, scaled particle theory, and thermodynamic flux analysis to explicitly incorporate the cell metabolism and the impact of the presence of macromolecular components on the nutrients diffusion. This framework was used to study two archetypical microbial communities (i) *Escherichia coli* and *Salmonella enterica* that cooperate with each other by exchanging metabolites, and (ii) two *E*. *coli* with different production level of extracellular polymeric substances (EPS) that compete for the same nutrients. In the mutualistic community, our results demonstrate that crowding enhanced the fitness of cooperative mutants by reducing the leakage of metabolites from the region where they are produced, avoiding the resource competition with non-cooperative cells. Moreover, we also show that *E*. *coli* EPS-secreting mutants won the competition against the non-secreting cells by creating less dense structures (i.e. increasing the spacing among the cells) that allow mutants to expand and reach regions closer to the nutrient supply point. A modest enhancement of the relative fitness of EPS-secreting cells over the non-secreting ones were found when the crowding effect was taken into account in the simulations. The emergence of cell-cell interactions and the intracellular conflicts arising from the trade-off between growth and the secretion of metabolites or EPS could provide a *local* competitive advantage to one species, either by supplying more cross-feeding metabolites or by creating a less dense neighborhood.

## Author summary

Microbial communities play a key role in biogeochemical cycles, bioremediation, and human health. In crowded microbial systems such as biofilms and cellular aggregates, the close proximity between individual cells reduces the free space for the nutrients diffusion. To model the heterogeneous nature of these microbial systems, we developed CROMICS, a framework that integrates the information about the metabolic capabilities of each individual cell as well as the size and location of cells and macromolecules in the medium. The interactions among the individuals arise naturally through competition for or the exchange of metabolites. We show how the presence of mutants and a reduced diffusion in crowded environments can perturb the local availability of nutrients and therefore modify the dynamics of a microbial community. The discovered mechanisms underlying the microbial interactions in crowded systems together with the developed framework represent a valuable starting point for future studies of the interplay of human microbiome and host metabolism, the pathogen invasion, and the evaluation of antibiotic effectiveness.

## Introduction

Microbial communities, such as biofilms, are involved in several processes ranging from beneficial bioremediation to the harmful fouling of industrial equipment, food contamination, and human chronic infections [1]. One of the main features of these communities is the close proximity among individuals, either because they are embeded in a biofilm matrix or due to physical restrictions of the system. The proximity of individuals facilitates the exchange of metabolic products and cell signaling [2], therefore promoting the emergence of cooperative interactions inside the community, but also promoting the competition for space and resources [3–5]. The presence of cells and other extracellular polymeric substances (EPS) in the biofilm matrix (i.e. macromolecules, DNA, polysaccharides, etc.) add another dimension of complexity to the system by creating a crowded environment, where the nutrient diffusion is unevenly reduced [6–11]. The crowding conditions are given by the volume fraction occupied by the cells and EPS. Changes in the availability of nutrients and solutes could have consequences on microbial dynamics, such as limiting the growth rate [12], reducing the effectiveness of antibiotic treatments [13], or modifying the protein expression levels of cells [14,15], and thus increasing the metabolic heterogeneity of the population. Herein, we investigate the influence of the crowding and environmental conditions as well as the metabolic variability of the species on the dynamics and interactions within microbial communities.

Simulating the competition/cooperation among microbial species that arises from the biosynthesis and secretion of metabolites and their diffusion in structured media has been successfully done through the application of the genome-scale metabolic models (GEMs) of the species [16–20]. The current computational frameworks proposed for modeling microbial systems oversimplify the crowding effect by assuming a homogeneous effective diffusion (*D*_*eff*,*met*_) *x* times slower than the diffusion in water, such that $D_{eff,met}=D_{met}^{0}/x$. For example in 3DdFBA [20], *D*_*eff*,*met*_ is a function of the local volume not occupied by cells of a similar size and shape. However, experimental evidence shows that *D*_*eff*,*met*_ is asymmetrical and depends on the local biofilm composition such as the abundance and size of EPS and cell species as well as the size of the diffusing molecule [6–11].

To provide a more realistic representation of the heterogeneous nature of microbial systems and study how the spatio–temporal-dependent crowding conditions affect the dynamic cooperation/competition among the individual cells, we developed a mechanistic framework for the **CRO**wding-**M**odeling of **I**n-silico **C**ommunity **S**ystems (CROMICS). CROMICS combines techniques such as individual-based modeling (IbM) and thermodynamic flux analysis (TFA) [21] to capture the metabolic variability within a population. For this, the metabolic activity of each cell is estimated under the local environmental conditions using GEMs. Additionally, the crowding effect is explicitly incorporated in the simulation by applying scaled particle theory (SPT) [22,23], thus *D*_*eff*,*met*_ in each region of the system is calculated as a function of the size and spatial distribution of cells and EPS with spherical shape.

As illustrative case study, we used CROMICS to simulate the co-growth of mutants of *Escherichia coli* and *Salmonella enterica* to demonstrate the competitive and mutualistic interactions among the species and subpopulations. We show how the crowding conditions favor the cooperation among individuals by focusing the exchange of metabolites in regions close the origin point, avoiding the metabolites leakage towards regions dominated by non-cooperative species. Thus, the fitness of cooperative mutant is enhanced as the crowding increased. Additionally, we investigated the crowding effect on the competition of two mutants of *E*. *coli* with different level of EPS production. The EPS secretion provided a competitive advantage to EPS-secreting mutants over non-secreting competitiors developing less dense structures, so that the amount of nutrients per mutant cell increased. In this case, the crowding modestly enhanced of the relative fitness of EPS-secreting cells over the non-secreting ones. These results give further understanding of the mechanisms underlying the interplay between the local composition and spatial organization in microbial communities and the inter/intra species interactions.

## Results and discussion

### CROMICS workflow

**Fig 1:**
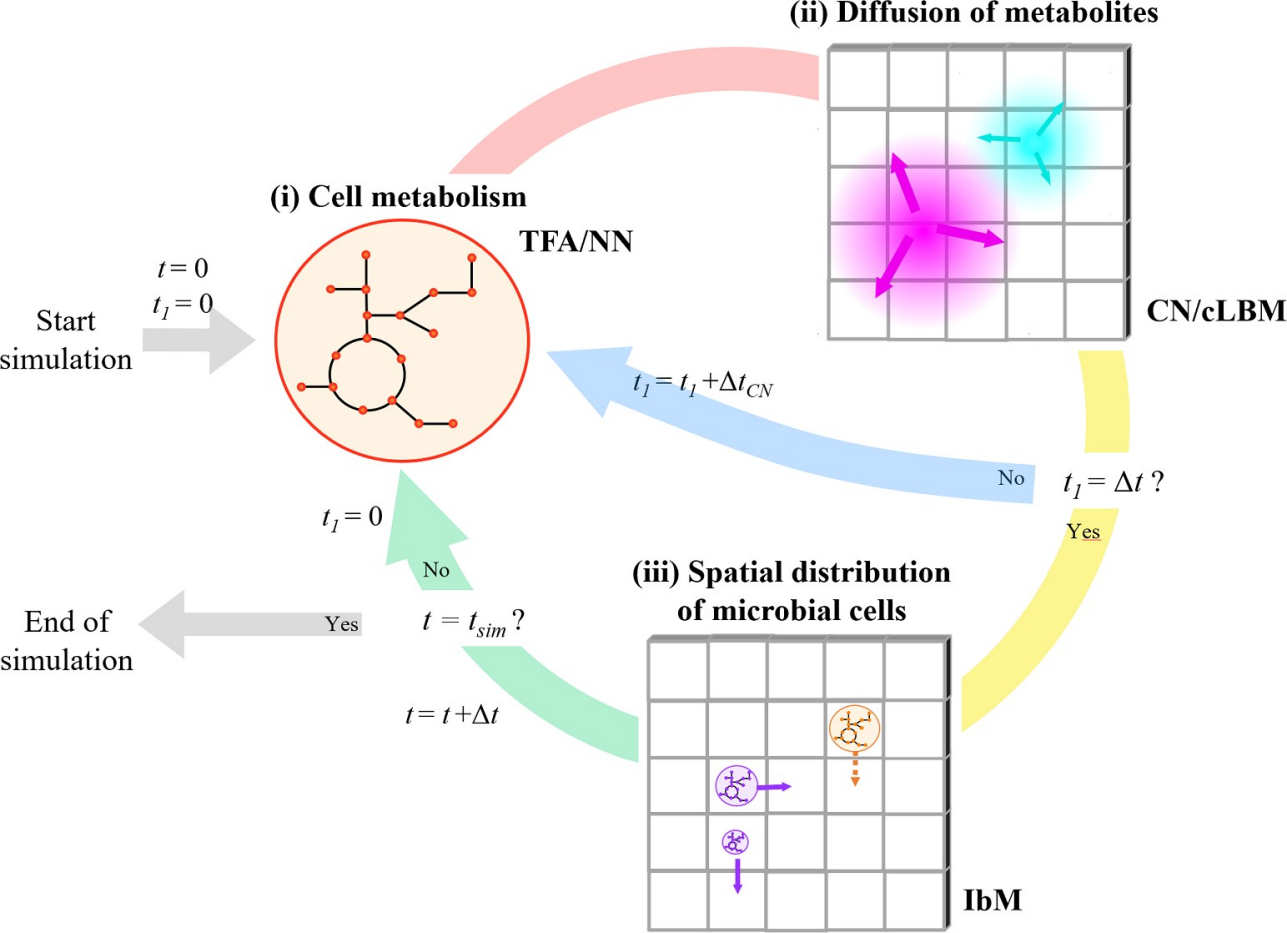
Workflow of CROMICS. The 2D or 3D system is divided in well-mixed boxes. At every time step Δ*t*, (i) the metabolic activity of the organisms is calculated using either thermodynamic flux analysis (TFA) or neural networks (NNs), then (ii) the diffusion of metabolites is approximated by the Crank-Nicolson method (CN) or crowding-lattice Boltzmann method (cLBM), and finally (iii) the behavior and spatial distribution of the microbial cells are computed using an individual-based modeling (IbM) approach. In step (iii), solid arrows indicate the cell motion, while dashed arrow indicates that cell division will take place. Since the uptake and diffusion of the metabolites are faster processes than the cell division and shoving (S1 Text IbM rules), steps (i) and (ii) can be simulated during a time *t*_*l*_ using a smaller time step Δ*t*_*CN*_ (Δ*t*_*CN*_ ≤ Δ*t*). Step (iii) is computed when *t*_*l*_ = Δ*t*. The simulation ends when the final simulation time *t*_*sim*_ is reached.

CROMICS allows the spatio-temporal modeling of microbial communities, wherein the heterogeneous aspects of the system, such as metabolic capabilities of the species and crowding conditions, can be incorporated. Thus, the effect of the spatial restrictions imposed by the presence of cells (and other macromolecules secreted to the medium) on the diffusion of nutrients and metabolites exchanged by the microorganisms arises naturally in the simulation. Here, the system is divided into small boxes or regions, where cells can uptake the nutrients locally available in a box. The simulation consists of three iterative steps (Fig 1). At every time step Δ*t*, (i) the growth rate and exchange metabolic fluxes of each microorganism are obtained from GEMs using either TFA or neural networks (NN) [24] specially trained for such purpose. NNs reduce the computational burden associated to the computation of the metabolic fluxes (see Methods). These metabolic fluxes are used to update the mass and volume of the cells as well as the amount (or concentration) of metabolites in each region. Then, (ii) the metabolites are allowed to diffuse to other regions, whose crowding conditions have changed due to the size increment of the cells. The effective diffusion is computed as $D_{eff,met}=\gamma_{met}^{-1}D_{met}^{0}$ [25], where the activity coefficient *γ*_*met*_ (calculated using SPT) represents the ratio between the total volume and the available volume for metabolite *met* in each region of the system. The metabolite diffusion in a 2D or 3D system can be computed using a crowding adaptation of either the semi-implicit Crank-Nicholson appoach [26] or the lattice Boltzmann method (cLBM) [27]. Finally, (iii) the cell division and re-distribution of species in the system is computed following IbM rules.

### Crowding conditions do not affect the species ratio convergence when there is not metabolic variability among individual cells

Spatio-temporal models are useful for studying and simulating the possible interdependence among microbial species that arises from sharing space and resources. We used CROMICS to simulate the growth of a mutualistic microbial community composed of *E*. *coli* K12 Δ*metB* and a methionine-secreting mutant of *S*. *enterica* (identified as meth^+^) in a 2D system. Ten bacterial spots with *E*. *coli* and meth^+^ were randomly inoculated. Each bacterial spot contained 3 x 10^−7^ g_DW_ spread over 200 individuals or metabacteria, where one metabacterium is collection of cells (see Methods. Community model 1). For simplicity, we will use the term cell to refer to metabacterium. The box height was set as Δ*z* = 0.18 mm, thus the initial crowding conditions (i.e. the volume fraction of the box occupied by cells) was on average 2%. Only lactose and O_2_ were continuously supplied to the system, maintaining an effective concentration of 2.92 mM and 0.21 mM in all boxes, respectively. The effective concentration is defined as the amount of metabolite per available volume (or volume not occupied by cells) in each box (Eq 2). *E*. *coli* is able to metabolize lactose under different oxygenation conditions and produce acetate and galactose as by-products, though this mutant is unable to synthesize methionine. On the other hand, meth^+^ can use both galactose and acetate (but not lactose) as a carbon source and synthesizes 0.5 mmol of methionine per gram of cell dry weight (g_DW_) [19]. The synergy between these two species relies on the mutual exchange of metabolites, i.e., *E*. *coli* use the methionine secreted by meth^+^, and the latter use the acetate and galactose produced by *E*. *coli*. Although at the beginning of the simulation there was not methionine (essential for the biomass synthesis) in the system, *E*. *coli* consumed lactose to satisfy its nongrowth ATP requirements. Small amounts of galactose and acetate are secreted as a consequence of lactose metabolism. Thus, *E*. *coli* started the cooperation with meth^+^ by releasing waste products obtained during the generation of energy for cell *maintenance*.

Two initial composition ratios of *E*. *coli*: meth^+^ were tested, i.e., 99:1 and 1:99. The results showed that regardless of the initial inoculum, the system converged in a species ratio of 76.3% ± 0.1% for *E*. *coli* and 23.7% ± 0.1% for meth^+^ after 14 h (when both microorganisms covered the whole system). This stability was achieved due to the cross-feeding interdependence that exists in the community, where one species cannot grow without the other. The convergence predictions of CROMICS agree well with both the experimental data and COMETS simulations [19], which use a spatio-temporal framework that approaches the dynamics of microbial populations (i.e., not individual cells like in CROMICS) by combining flux balance analysis and diffusion (S1 Fig). Unlike CROMICS, COMETS assumes that the metabolite diffusion coefficient is constant, meaning that cells do not affect the metabolite diffusion. To determine if the crowding conditions affected the CROMICS predictions in this case study, we simulated the system again assuming volumeless cells and $D_{eff,met}=D_{met}^{o}$.

**Fig 2:**
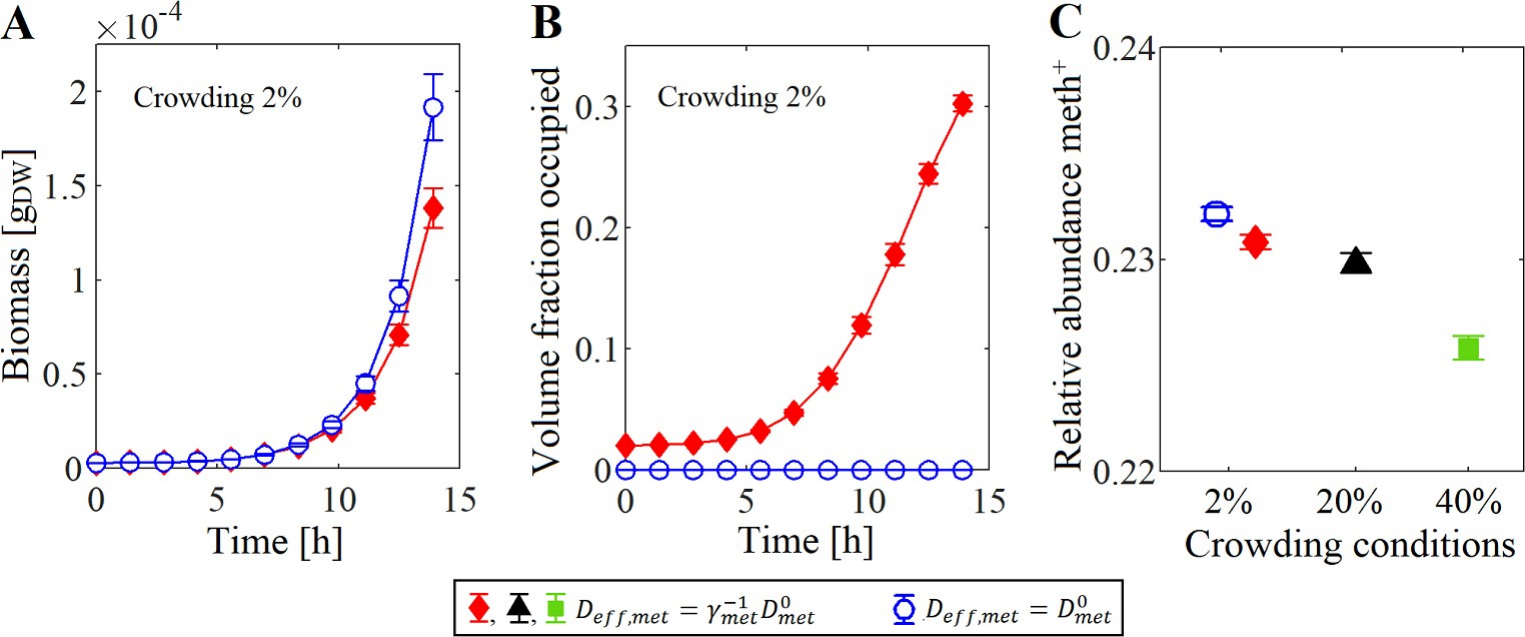
Dynamics of the mutualistic community *E*. *coli* Δ*metB*: methionine-secreting mutant of *S*. *enterica* (meth^+^) with an initial composition of 99:1. (A) Total biomass. (B) The average volume fraction occupied by the cells (i.e. crowding conditions) in their corresponding box. (C) The species ratio convergence of meth^+^. Three initial crowding conditions were tested: 2% (using a box height Δ*z* = 0.18 mm), 20% (Δ*z* = 0.018 mm), and 40% (Δ*z* = 0.009 mm). Closed symbols represent simulations where the crowding effect was taken into account ($D_{eff,met}=\gamma_{met}^{-1}D_{met}^{0}$), while open symbols represent simulations where the crowding was neglected ($D_{eff,met}=D_{met}^{0}$). Error bars show the standard deviation in five independent simulations.

For an initial composition of 99% *E*. *coli* and 1% meth^+^, the results showed that at the beginning of the simulation, when the cells occupied less than 10% of their current box volume, there is not difference in the total biomass computed in both simulations. However, such biomass difference increased as the system became more crowded, that is when cells grew and occupied more than 10% of the box volume (Fig 2A and 2B). The biomass accumulated in the system was lower in the simulations where the crowding effect was taken into account (Fig 2A). This may be because the metabolite diffusion decreased as the system became more crowded, making it difficult the re-distribution and access to the metabolites exchanged by the cells (methionine, acetate and galactose). Nevertheless, a similar relative abundance of the species was obtained in both simulations (Fig 2C).

To determine if the initial crowding conditions have an impact on the relative species abundance, we artificially increased the crowding (i.e. volume fraction occupied by the cells, *V*_*cell*_/*V*_*box*_) by reducing the height Δz of the system, thus, the box volume (*V*_*box*_ = Δ*x*Δ*y*Δ*z*) also decreased. This is similar to have the microbial community in a 2-plate chamber of Δz in height. Three initial crowding conditions were tested: 2% (Δ*z* = 0.18 mm), 20% (Δ*z* = 0.018 mm), and 40% (Δ*z* = 0.009 mm). For an initial ratio *E*. *coli*: meth^+^ of 99:1, the results confirmed that the biomass achieved in the simulation decreased as the initial crowding increased, because the metabolites diffuse slower from the production point. Nevertheless, the crowding conditions did not affect the convergence ratio of the species (Fig 2C). That is the convergence ratio 76% for *E*. *coli* and 24% for meth^+^ was determined by the amount of the metabolites exchanged by the species.

**Fig 3:**
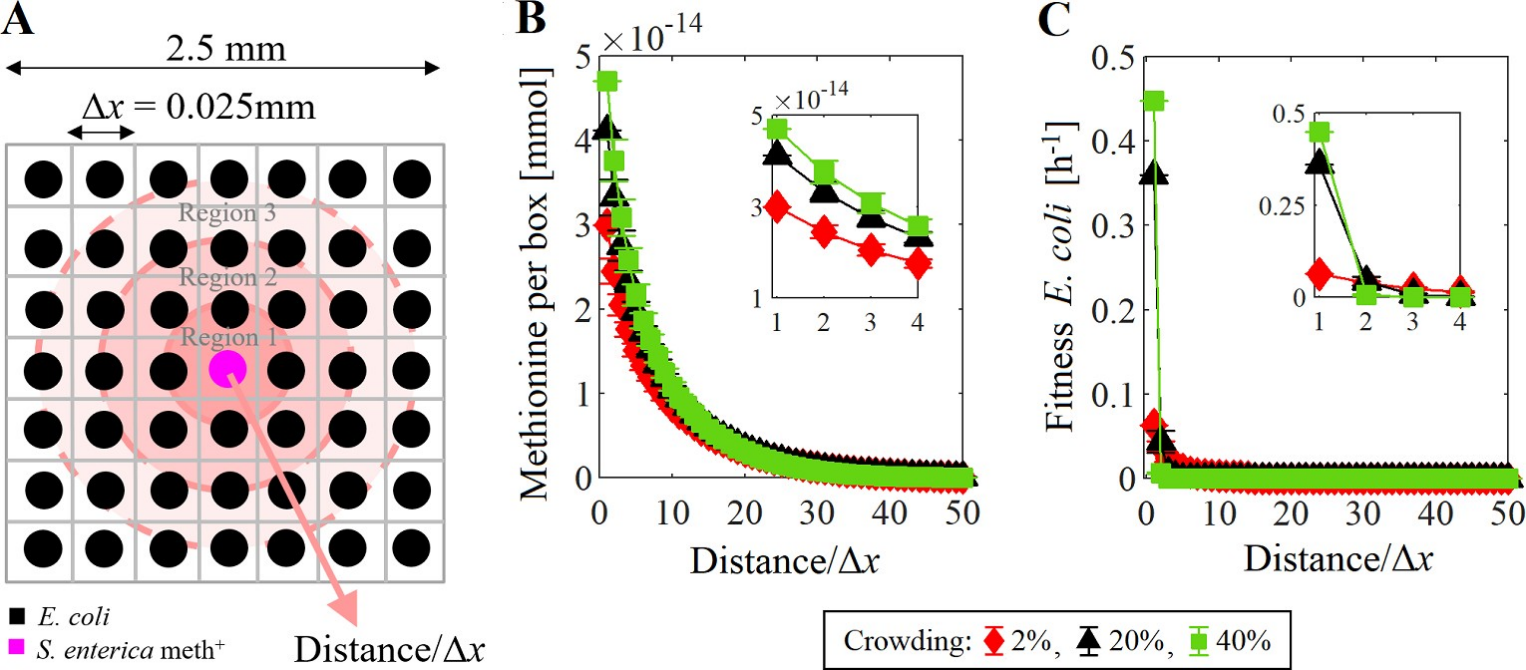
Availability of methionine and fitness as a function of the distance from the production point in crowded environments. The microbial community was composed by *E*. *coli* Δ*metB* and methionine-secreting mutant of *S*. *enterica*. (A) Schematic representation of the spatial distribution of the species. (B) Average amount of methionine available per box (inset) enlarged graph assuming that *E*. *coli* cells are inactive, and (C) fitness of active *E*. *coli* cells (inset) enlarged graph at different crowding conditions. Error bars show the standard deviation in the system (one independent simulations).

In crowded environments the access to the exchanged metabolites depends on the distance from the production to consumption point. As an example, we simulated the production and diffusion of methionine in a small system of 2.5 mm by 2.5 mm divided in boxes of Δ*x* = 0.025 mm per sides (Fig 3A), and under three different crowding conditions: 2% (Δ*z* = 0.18 mm), 20% (Δ*z* = 0.018 mm), and 40% (Δ*z* = 0.009 mm). The system was filled with inactive cells (i.e. they cannot consume methionine nor grow), and only one meth^+^ cell was placed in the center. The mass of each cell was set to 7.5 x 10^−10^ g_DW_. The effective concentration of O_2_, lactose and acetate in the system was fixed to 0.21, 2.92, and 10 mM, respectively, that is meth^+^ does not depend on the acetate produced by *E*. *coli*. The system was divided in areas or regions delimited by concentric circles of radii Δ*x*, 2Δ*x*,…, 50Δ*x*. The methionine available per box in the different regions was obtained by averaging the amount of methionine in the boxes whose center is located between two concentric circles. A snapshot of the average abundance of methionine per box after 6 min of simulation showed that the amount of methionine retained or concentrated at Δ*x* = 0.025 mm from meth^+^ increased as the crowding increased (Fig 3B). Even more, simulations where the inactive cells were replaced by active *E*. *coli* revealed that the fitness of cells located at Δ*x* from meth^+^ were higher in more crowded systems, e.g. at 40% (Fig 3C), that may be due to a higher methionine retention in such region. However when the crowding was set to 40%, only *E*. *coli* cells within a radial distance of 2Δ*x* had access to the methionine produced by meth^+^, allowing them to grow (Fig 3C), while in less crowded systems (20% and 2%), the methionine can diffuse further (5Δ*x* and 14Δ*x*, repectively) before being almost depleted by the *E*. *coli*. This suggested that the crowding conditions can limit the area of microbial interaction. Therefore, crowding effect could become more important in heterogeneous systems with metabolic variability among individuals that compete for the same nutrients as shown in the next section.

### Crowding conditions enhance the fitness of cooperative mutants

Metabolic variability among the individuals of a population of the same species may arise due to differences in protein expression levels or gene mutation [28,29]. In many cases, the (over)production of cross-feeding metabolites comes at the expense of microorganism growth [30]. For example in *S*. *enterica*, the secretion of methionine reduces the fitness (expressed in terms growth rate) of the cell inasmuch as more nutrients and proteome resources are invested for the synthesis of the amino acid. The comparison of the maximum growth rate computed by TFA for two *S*. *enterica* subpopulations revealed that a methionine-secreting mutant (meth^+^) reduces its growth rate in 7% compared to the wild type non-methionine-secreting (meth^-^). Here, we analyzed the crowding effect on the fitness of cooperative mutants and the competition among two subpopulations.

Following our previous case study, we simulated the co-growth of *S*. *enterica* and *E*. *coli* Δ*metB*, using an initial species ratio of 50:50 and assuming that cells occupied 40% of the box volume. *S*. *enterica* species consisted of subpopulations meth^-^ and meth^+^. Twenty bacterial spots were inoculated in the system with an equal number of individuals of *E*. *coli* and *S*. *enterica*, but only one spot (identified as colony A) contained meth^+^, we tested three different initial number of meth^+^ cells 70, 60, and 50 (corresponding to a relative abundance of 8.8%, 7.5%, and 6.3%, respectively). All cells were randomly allocated in the bacterial spots. See the model setup in Methods. Community model 1.

**Fig 4:**
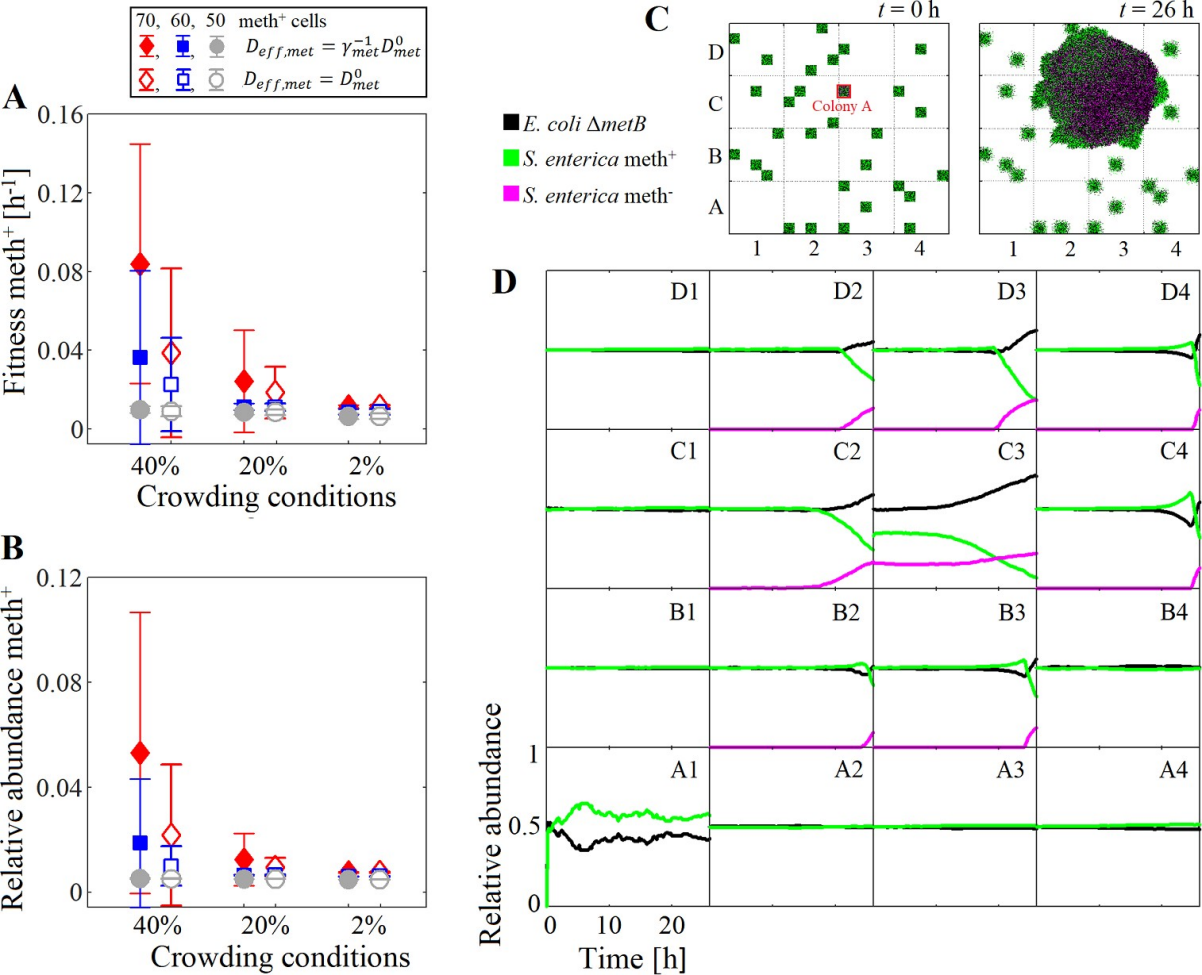
Dynamics of the community *E*. *coli*—*S*. *enterica* with metabolic variability. (A) Fitness and (B) relative abundance of meth^+^ computed after 26 h. Three different initial crowding conditions were tested: 2% (Δ*z* = 0.18 mm), 20% (Δ*z* = 0.018 mm), and 40% (Δ*z* = 0.009 mm), with initial frequencies of 70, 60, and 50 meth^+^ cells. Closed symbols represent simulations where the crowding effect was taken into account, while open symbols represent simulations where the crowding was neglected. (C) Snapshot of the spatial distribution of cells in one simulation repetition at *t* = 0h and *t* = 26 h. Only 1 out of 20 bacterial spots, colony A, where inoculated with 70 meth^+^ cells, the initial crowding conditions were set as 40%. (D) Dynamics of the relative abundance of the species predicted in 20 different regions of the system (the time is represented on the *x* axis of each quadrant, while the relative abundance is on the *y* axis). Error bars show the standard deviation in five independent simulations.

The comparison of the average fitness or growth of meth^+^ (Eq 10) showed a positive frequency-dependent selection for meth^+^, where its fitness increased as meth^+^ was more common in the initial set up of the system (Fig 4A). This is because more methionine can be produced when the abundance of meth^+^ was higher, and therefore *E*. *coli* can grow faster and synthesize more acetate and galactose for both *S*. *enterica* subpopulations.

To determine if the crowding have an impact on meth^+^ fitness, we tested four initial crowding conditions: 2% (Δ*z* = 0.18 mm), 20% (Δ*z* = 0.018 mm), and 40% (Δ*z* = 0.009 mm). Results showed that for the same initial meth^+^ frequency, the fitness of meth^+^ was enhanced when the crowding increased. The diffusion of metabolites is reduced in crowded media, such as colonies or biofilms, meaning that crowding can minimize the leakage of exchanged metabolites towards regions dominated by competitors such as meth^-^, favoring in this way the proliferation of cooperative mutants. In comparison, meth^+^ reached a lower fitness and relative abundance when the crowding effect was neglected in the simulations (open symbols in Fig 4A and 4B), because the exchanged metabolites can easily escape from origin point when the colonies are made up of volumeless cells. Thus, for the initial frequency tested, the crowding conditions favor the invasion of cooperative mutants over non-cooperative ones.

For an initial frequency of 70 meth^+^ cells and under 40% of initial crowding conditions (Δ*z* = 0.009 mm), the snapshot of the spatial distribution of the species after 26 h (Fig 4C, left) showed that colony A proliferated due to the cooperation between the *E*. *coli* and meth^+^. Colony A eventually expanded and incorporated neighboring colonies formed with meth^-^. Meth^-^ cells benefitted from the cross-feeding resources, and their density at the peripheral of the colony increased (Fig 4C). The exclusion of non-cooperative species to the colony periphery has been experimentally observed for example in *Vibrio cholerae* biofilms [31].

A close inspection of the dynamics of the species abundance in different regions of the system revealed that meth^-^ became more abundant than *E*. *coli* in regions near colonies A. This can be seen, for example, in quadrant C4 of Fig 4D, where acetate and galactose were available due to their diffusion from the source colony. However, once meth^+^ appeared in the quadrant due to the expansion of colony A, the abundance of *E*. *coli* increased and surpassed the *S*. *enterica* mass fraction. The high variability found in the species profile suggests that each region can respond differently to a medium stimulus due to the local interactions among species and subpopulations.

These results showed that a cooperative mutant can easily dominate the system when the mutation provides a competitive advantage. In this case, this advantage occurs through the cooperation with other species, even if the cell sacrifices its own growth rate in favor of the synthesis of metabolites. The successful invasion of meth^+^ in a community formed by *E*. *coli* and *S*. *enterica* WT has been experimentally observed in spatially structured environments [32]. While the cooperation among species is favored in structured environments where the short distances between the cells facilitates the exchanged metabolites [33,34], our results showed that the crowding conditions may enhance the fitness of cooperative species by reducing the diffusion and leakage of the metabolites. This suggests that the microbial dynamics depends on the local production level of cross-feeding metabolites (i.e. on initial frequency of the producing species) as well as the crowding conditions (Fig 4).

### Crowding conditions have a modest effect on the competition among species

Frequently, microbial communities are composed of a mixture of species, each one with its own metabolic capacities, cell size and shape. Experimental measurements indicate that the effective diffusion of chemical species (metabolites, antibiotics, etc.) not only depends on their location in the biofilms but also on the species composition of the biofilm [6–11]. This could be related to the structure formed by the microbial species and EPS molecules of different sizes and shapes.

**Fig 5:**
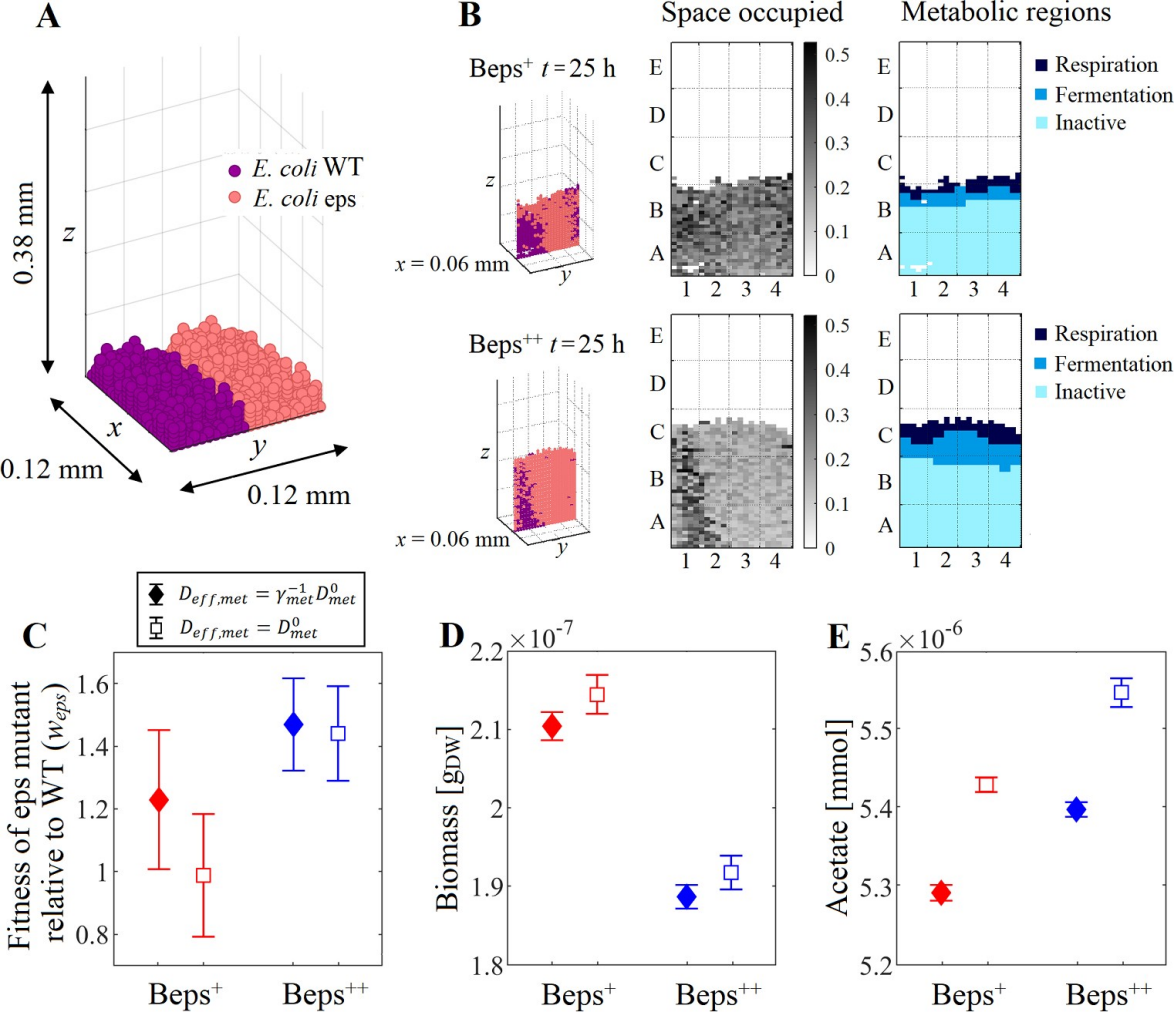
Dynamics of the biofilm growth of two *E*. *coli* species with different EPS production level. (A) Spatial distribution at *t* = 0 h of *E*. *coli* cells WT and eps mutant (eps^+^ or eps^++^). (B) Snapshots of the space occupied only by the cells and the metabolic regions in the vertical layer (0.06 mm, *y*, *z*) of biofilms Beps^+^ and Beps^++^ at 25 h. (C) Fitness of the eps mutant relative to WT. (D) Total biomass and (E) the acetate produced by the microbial community at 25 h. Closed symbols represent simulations where the crowding effect was taken into account ($D_{eff,met}=\gamma_{met}^{-1}D_{met}^{0}$), while open symbols indicate simulations where the crowding was neglected ($D_{eff,met}=D_{met}^{0}$). Error bars show the standard deviation in five independent simulations.

The polymer secretion has been previously identified as a competitive advantage in multi-species biofilm simulations by assuming $D_{eff,met}=D_{met}^{0}$ [3]. In this section, we investigated the crowding effect on the competition of two species with different EPS production level. The growth of two *E*. *coli* biofilms composed by WT cells and EPS-secreting mutants were used as case studies. In biofilm Beps^+^, the mutants secreted 0.11 g g_DW_^-1^ of EPS (identified as eps^+^); while in biofilm Beps^++^, the mutants secreted 0.43 g g_DW_^-1^ of EPS (identified as eps^++^). The mutants invest part of their resources in the EPS production, so that eps^+^ and eps^++^ reduce their growth rate in 10% and 30% compared to WT, respectively. Due to the production and accumulation of EPS, a lower number of mutant cells can be allocated in each box. Thus, a metabacterium of WT, eps^+^, eps^++^ contained 27, 20, and 12 cells, respectively, and the maximum metabacterium mass $M_{max,{eps}^{+}}$ was then computed as 0.74*M*_*max*,*WT*_, while $M_{max,{eps}^{++}}$ as 0.45*M*_*max*,*WT*_. In both Beps^+^ and Beps^++^, WT cells were initially located at the left side of the biofilm, while mutants occupied the right side (Fig 5A). Glucose and O_2_ was supplied to the system. The cell size and macromolecules of 400 Da or greater were explicitly considered in the CROMICS simulations. All other molecules (≤ 400 Da) were considered volumeless. See the model setup in Methods. Community model 2.

Although eps^+^ and eps^++^ sacrificed their own growth in favor of EPS synthesis, the fitness of the mutant relative to WT, *w*_*eps*_ = *Fitness*_*eps*_/*Fitness*_*WT*_ (Eqs 10 and 11), computed in both biofilms showed that EPS secretion provided competitive advantage to mutants over WT (Fig 5C). By secreting EPS to the medium, the mutants created a less dense cell structure where a lower number of eps^+^ or eps^++^ cells shared the same box or region (which was taken into account by reducing the mass threshold to carry out the cell division $M_{max,{eps}^{+}}$ and $M_{max,{eps}^{++}}$ compared to WT), so more nutrients are available per g_DW_ of mutant. Even more, the crowding conditions hindered the diffusion of nutrients towards bottom layers of the biofilm, favoring in this way the growth of species located at the biofilm surface. In our case studies, EPS molecules pushed the mutant cells up, so mutants can reach the upper biofilm layers more easily than WT, and thus, have access to the nutrients retained by the crowding conditions. In comparison, the increment in the EPS production of eps^++^ compared to eps^+^ enhanced the relative fitness of mutant in the biofilm (Fig 5C). This may be because eps^++^ accumulated more EPS that allowed them to reach higher regions rich in nutrients (near the top boundary) where eps^++^ could grow faster than WT. Nevertheless, the higher fitness cost paid by eps^++^ for the production EPS caused that total biomass accumulated in the biofilm Beps^++^ was lower than in Beps^+^ (see inset of Fig 5D).

When the volume of both cells and EPS were neglected in the simulations (making the diffusion coefficients $D_{eff,met}=D_{met}^{o}$) there was more free space in the system for the entry of nutrients, thus the community reached a higher biomass at 25 h than that obtained in simulations where the crowding effect was taken into account (Fig 5D). However, the relative fitness of both eps^+^ and eps^++^ were lower than that obtained in the simulations where the size of the biofilm components were considered. In this case, the crowding conditions have only a modest effect on the competition between EPS-secreting mutants and WT, suggesting that when the nutrients are supplied externally (from the top boundary, and not produced by a species partner) other factors such as the biofilm cell structure (i.e. the spacing between cells) have a greater impact on the microbial dynamics of the microbial community.

The space occupied by cells on the left and right sides of both biofilms at 25 h changed as a result of the difference in the EPS production between WT and mutants (Fig 5B). Due to the different cell structures reached in both biofilms Beps^+^ and Beps^++^, the cells were exposed to a different concentration gradient of nutrients that could shift their metabolisms. Phenotypes of both WT and mutants were identified using their metabolic fluxes (Eq 9). Three phenotypic regions were identified in the biofilms: (i) on the superficial layers where O_2_ is still available, both WT and mutant cells activated the respiratory pathways, (ii) cells in intermediate layers opted for fermentative pathways producing large amounts of acetate, and (iii) inactive cells appeared at bottom layers where both glucose and O_2_ were depleted (Fig 5B). Larger inactive regions were found in both biofilms in regions dominated by WT. This can be explained by the denser structure formed by WT compared to mutants, which made it difficult to move and replenish nutrients from the top boundary (Fig 5B). In particular, the lower availability of O_2_ in the biofilms caused the cells to shift to a fermentative metabolism. The emergence of one metabolic phenotype or another depending on the nutrient availability can lead to the secretion of different amount of metabolites, e.g. the acetate produced in the biofilm Beps^+^ was lower than in Beps^++^ (Fig 5E), which could change the cross-feeding interactions if one of the species in the system depends on the acetate availability as in our previous case study *E*. *coli*—*S*. *enterica*. Moreover, the production and accumulation of acetate in the system may have an additional effect on the community dynamics. For example, high concentration of acetate (and other organic acids) inhibits the microbial growth due to the perturbation of the anion balance and the uncoupling effect (where cell have to expend energy to expel H^+^ and maintain the membrane potential) [35], which is not considered in CROMICS simulations.

As shown above, the heterogeneous nature of microbial communities is also reflected in the *local* cell structure of the system, meaning the spatial arrangement of cells and EPS components, which can favor the emergence of certain phenotypes (and therefore the production of a metabolite) or even provide a competitive advantage to one species over the others. In communities competing for the same resources, such as Beps^+^ or Beps^++^, the less dense structure reached by the mutants allowed them to access regions with richer nutrients, therefore win the competition to WT. In this simplified example, the cell structure was determined by the EPS production, though other factors could affect the configurations, such as the shape of the cells, the production of different type of EPS molecules, the electrostatic interactions between the molecules that prevent close proximity, etc.

## Conclusion

Modifications to local environments that occur by the secretion of shared resources (exchanged metabolites and EPS molecules) can provide a competitive advantage to certain species, though this imposes resource allocation conflicts at the cellular level that could lead to a reduction of the growth rate. The crowding conditions can boost such competitive advantage by restricting the area of interactions in a microbial community. We showed how the crowding enhanced the fitness of cooperative mutants by reducing the leakage of the exchanged metabolites from the production point. In the case where nutrients were supplied externally to a biofilm community (instead of being produced by partner species), the fitness of an EPS-secreting mutant relative to non-secreting cells was modestly enhanced by the crowding effect, suggesting that the formation of a less dense structure due to EPS accumulation had a greater effect on the competition between these species, where the EPS-secreting mutant was the winner. Further studies to identify the specific scenarios wherein the crowding effect becomes important to microbial dynamics could help to simplify this layer of complexity.

Modeling approaches like CROMICS can contribute to efforts to bridge the gap between the modeling of single cell metabolisms and whole populations. The versatility of these approaches allows one to explore the interplay between the physical restrictions imposed by cell growth and macromolecule secretion that negatively affect the nutrients diffusion and the dependence of the cell metabolism on the local availability of nutrients in microbial systems.

## Methods

The spatio-temporal microbial modeling developed in CROMICS is an iterative process that integrates information about (i) the cell metabolism, (ii) the diffusion of metabolites, and (iii) the redistribution of individual cells in the system (Fig 1). CROMICS requires an input of the parameters (e.g. diffusion coefficients, Michaelis-Menten constants) and the initial set up of the system (GEM, initial seed of cells and metabolites). The system is discretized on a regular lattice, with meshing sizes Δ*x*, Δ*y*, and Δ*z* along the spatial coordinates. 2D systems are simulated by assuming a monolayer of rectangular (or cubic) prism boxes.

The metabolite diffusion process and the distribution of microbial species are simulated on two different lattices identified as IbM and CN lattice, respectively. For notation simplicity, the same lattice size (Δ*x*) is used to describe the spatio-temporal distribution of both cell species and metabolites. However, a coarse-grained discretization can be applied for the metabolites diffusion (see S1 Text Coarse-grained considerations). Assuming cells of spherical shape, then Δ*x* is two times the maximum cell radius *R*_*max*,*cell*_ before cellular division occurs.

In CROMICS, the spatio-temporal distribution of the nutrients and microbial species are computed for each discrete time step Δ*t* until the final simulation time *t*_*sim*_ is reached. In microbial systems, the diffusion of metabolites is faster than the cellular processes (e.g. cell division and shoving). Shoving process is described in S1 Text IbM rules. Therefore, the processes (i) and (ii) depicted in the CROMICS workflow (Fig 1), i.e. cell metabolism and metabolite diffusion, can be simulated using a smaller time step Δ*t*_*CN*_, while the cellular processes (iii) can be computed using a longer Δ*t*, i.e. Δ*t*_*CN*_ ≤ Δ*t*. A description of the CROMICS framework is given below for a 3D system, and the extension to 2D systems is done in a straightforward way. The setup of the case studies simulated (*E*. *coli—S*. *enterica* consortium and *E*. *coli* biofilm) are given in Case studies.

### (i) Metabolism and cell growth

The interactions between a cell and the medium contained in the same box *ijk* during a time Δ*t*_*CN*_ are determined by the metabolic capabilities of the microorganism and the exchange rate of metabolites. Cells can uptake nutrients from the local box *ijk*. If the nutrient uptake is mediated by protein membranes (i.e. active transport), the maximum uptake flux *v*^*U*^_*f*,*ex*,*met*_ is bounded by the Michaelis-Menten kinetics.


$$v_{f,ex,met}^{U}=\frac{V_{M,met}C_{eff,met}(ijk,t)}{K_{M,met}+C_{eff,met}(ijk,t)}.$$ (1)

While for passive transport of metabolites, the uptake rate is constrained by $v_{f,ex,met}^{U}=\min\left(V_{M,met},\rho_{met}(ijk,t)/M_{cell}(t)\Delta t\right)$, where *ρ*_*met*_ is the (extracellular) amount of metabolite *met* in the box *ijk* [mmol], and *M*_*cell*_ is the cell mass at time *t* [g_DW_]. The values of the maximal uptake flux (*V*_*M*,*met*_) [mmol g_DW_^-1^ h^-1^] and the Michaelis-Menten constant (*K*_*M*,*met*_) [mmol L^-1^] can be obtained from experimental data and/or databases (e.g. BRENDA database). *C*_*eff*,*met*_ [mmol L^-1^] represents the effective concentration of the metabolite in the box *ijk*, given by

$$C_{eff,met}(ijk,t)=\frac{\rho_{met}(ijk,t)}{10^{6}V_{box}}\gamma_{met}(ijk,t).$$ (2)

where the volume of a box is *V*_*box*_ = Δ*x*Δ*y*Δ*z* [mm^3^], 10^6^ is the conversion factor from mm^3^ to L. Assuming that both cells and metabolites are hard spheres of different radii *R* [mm], the activity coefficient *γ*_*met*_ (i.e. the ratio between the box volume and the available volume for *met* in the box *ijk*) can be estimated by SPT [22,23]

$$\ln\gamma_{met}=-\ln(1-S_{3})+(\frac{6S_{2}}{1-S_{3}})R_{met}+(\frac{12S_{1}}{1-S_{3}}+\frac{18S_{2}^{2}}{{\left(1-S_{3}\right)}^{2}})R_{met}^{2}+\left(\frac{8S_{0}}{1-S_{3}}+\frac{24S_{1}S_{2}}{{\left(1-S_{3}\right)}^{2}}+\frac{24S_{2}^{3}}{{\left(1-S_{3}\right)}^{3}}\right)R_{met}^{3},$$ (3)

where the variable *S*_*x*_ is given by

$$S_{x}=\frac{\pi }{6V_{box}}\left(\sum_{l}^{macromolecules}\frac{\rho_{l}N_{A}}{10^{3}}{\left(2R_{l}\right)}^{x}+\sum_{l}^{cells}{\left(2R_{cell}\right)}^{x}\right),\;x=0,1,2,3.$$ (4)

For simplicity, the index *(ijk*,*t)* has been dropped from *γ*_*met*_ and *S*_*x*_. *k*_*B*_ represents the Boltzmann constant, and *N*_*A*_ is Avogadro’s constant. The factor 10^3^ in Eq 4 indicates the conversion from mol to mmol. Eq 4 was modified for metabolites able to penetrate the cell membrane, see details in S1 Text SPT considerations. Note that if the size of the metabolites is considered negligible compared to the size of cells (i.e. *R*_*met*_ = 0), the last three terms disappear from the right hand side of Eq 3. Thus, 1/*γ*_*met*_ is given by the volume fraction not occupied by cells.

Once the uptake flux limits *v*^*U*^_*f*,*ex*,*met*_ are set based on the effective nutrient concentration in the medium (Eqs 1 and 2), then the growth rate *ν*_*bio*_ and exchange flux of metabolites to/from the cell *ν*_*f*,*ex*,*met*_ [mmol g_DW_^-1^ h^-1^] can be calculated by TFA (which involves mass conservation and thermodynamics constraints) [21] or alternatively by NN [24]. See details in S1 Text Metabolic flux estimations. Other stoichiometric models and constraints can also be applied. When no feasible flux solution was found due to the starvation conditions, the cell was allowed to shrink with a rate *v*_*shrinkage*_ to satisfy the cell maintenance requirements, i.e., *ν*_*bio*_ = *v*_*shrinkage*_. *ν*_*bio*_ and *ν*_*f*,*ex*,*met*_ were used to update cell mass *M*_*cell*_ and the amount of metabolite *ρ*_*met*_ in each box for the next time *t* + Δ*t*, so that

$$\rho_{met}(ijk,t+\Delta t)=v_{f,ex,met}(ijk,t)M_{cell}(ijk,t)\Delta t+\rho_{met}(ijk,t),$$ (5)

$$M_{cell}(ijk,t+\Delta t)=v_{bio}(ijk,t)M_{cell}(ijk,t)\Delta t+M_{cell}(ijk,t).$$ (6)

We assume that the cell volume is proportional to *M*_*cell*_ and the specific volume for species *sp* (*υ*_*sp*_), therefore the cell radius *R*_*cell*_ at time *t*+Δ*t* is given by

$$R_{cell}(ijk,t+\Delta t)={\left(\frac{3}{4\pi }M_{cell}(ijk,t+\Delta t)\upsilon_{sp}\right)}^{1/3}.$$ (7)

The increase in the cell size (expressed in terms of *R*_*cell*_) modifies the crowding conditions prevailing in box *ijk* such that the activity coefficient of the metabolites *γ*_*met*_ will change in accordance with Eq 3. The direct relationship between *γ*_*met*_ and *C*_*eff*,*met*_ (Eq 2) indicates that the effective concentration experienced by a cell is affected by: (i) the time-dependent (local) crowding conditions, and (ii) the changes in the amount of *met* due to the consumption/production of the metabolite or the entrance of new molecules from neighboring boxes. Thus, *C*_*eff*,*met*_ (Eq 2) provides the link between the diffusional problems found in biofilms caused by crowding conditions and its effect on cell metabolism and microbial growth.

### (ii) Diffusion of metabolites in a crowded system

In biofilms, as in any other crowded system, the diffusion of metabolites is negatively affected by the presence of microbial cells and other solid components that reduce the available space for molecular motion. The re-distribution of the metabolites across the system can be computed by solving the diffusion equation

$$\frac{\partial \rho_{met}}{\delta t}=\nabla ∙(D_{eff,met}{\nabla \rho }_{met}),$$ (8)

The effective diffusion in each box is given by $D_{eff,met}=\gamma_{met}^{-1}{(ijk)D}_{met}^{0}$ [25], where $D_{met}^{o}$ is the diffusion coefficient in water and *γ*_*met*_ is calculated using SPT (Eq 3).

In this paper, the diffusion equation is solved by applying a crowding version of either a semi-implicit Crank-Nicholson (CN) approach [26] or the Lattice Bolzmann Method [27], see details in S1 Text Metabolite diffusion. cLBM allows the computation of the mean squared displacement (MSD) of the molecules, which is useful for studies of anomalous diffusion [36].

### (iii) Spatial distribution of the microbial cells

The behavior of a microbial cell and its interactions with neighboring cells are simulated by IbM rules that, as a whole, will determine the evolution and spatial distribution of the microbial system. Cell properties of each individual, such as the box position *ijk*, *M*_*cell*_, *R*_*cell*_ (Eqs 6 and 7, respectively) and the metabolic phenotype *Phen* are tracked at every time step. *Phen* is computed based on the exchange metabolic fluxes obtained from TFA/NN and a threshold value *θ* = 10^−4^ mmol g_DW_^-1^ h^-1^.


$$Phen=\left\{ \begin{array}{cc} 1 & if\;v_{f,ex,met}<\theta \\ 2 & if\;v_{f,ex,met}\geq \theta \end{array}.\right.$$ (9)

Symmetric or asymmetric cell divison can take place when *M*_*cell*_ reaches the maximum dry mass *M*_*max*,*sp*_. The daughter cell is allocated in random neighboring box, next to the mother cell that remains in the current site *ijk*. When all neighboring boxes are occupied by cells, then daughter cell will shove/displace neighbour cells. Alternatively, the cell dies under starvation conditions when *M*_*cell*_ is less than the minimal dry mass threshold *M*_*min*,*sp*_. Additionally, the random or biased walk of cells can also be incorporated in the simulations. See details in S1 Text IbM rules.

CROMICS framework was implemented in Matlab R2018b. The metabolic fluxes can be computed using either NN or TFA. TFA code is available at https://github.com/EPFL-LCSB/mattfa. The metabolic flux samples required for the NN training were computed using TFA with CPLEX solver, and the training was performed using the Matlab Deep Learning Toolbox.

### Case studies

#### Community model 1: *E*. *coli* K12 Δ*metB* and *S*. *enterica*

The community composed of *E*. *coli* K12 Δ*metB* and the *S*. *enterica* methionine-secreting mutant was simulated on a 2D system with a CN-lattice of 200 x 200 boxes (S1 Fig) [19]. Each CN-box was composed of sides Δ*x*_*CN*_ = 0.05 mm and a height of Δ*z*_*CN*_, which was selected to get the desired initial crowding conditions (i.e. the volume fraction occupied by the cells, *V*_*occ*_). Thus, the parameter Δ*z*_*CN*_ was computed as $\Delta z_{CN}=M_{cell}\upsilon_{sp}/(V_{occ}\Delta x_{CN}^{2})$. The CN-lattice is superposed on an IbM-lattice of 400 x 400 boxes of Δ*x*_*IbM*_ = 0.025 mm and a height Δ*z*_*IbM*_ = Δ*z*_*CN*_, wherein each CN-box contains four IbM-boxes. The time step Δ*t* was set as Δ*t* = Δ*t*_*CN*_ = 1.25 s. Ten bacterial spots were randomly inoculated with *E*. *coli* and *S*. *enterica* in a proportion of either 1:99 or 99:1. One bacterial spot contained 400 metabacteria allocated in 400 IbM-boxes. A metabacteria is a collection of bacteria of the same species with the same size and metabolic capabilities that move (diffuse) in the same direction. The initial mass of each metabacteria was randomized using a normal distribution with mean 4.89 x10^-13^ g_DW_ and standard deviation 1.32 x10^-13^ g_DW_ [37] valid for single cells, and this was multiplied by the number of cells in a metabacteria (*metaB*). *metaB* was computed as a function of the box height selected Δ*z*_*IbM*_, i.e. *metaB* = 0.52Δ*x*_*IbM*_Δ*y*_*IbM*_Δ*z*_*IbM*_/(*M*_*max*,*sp*_*υ*_*sp*_), where the factor 0.52 represents the densest packing of spherical cells in a square lattice. In total, each bacterial spot contained a biomass of 3 x 10^−7^ g_DW_. The volume of the cells was assumed to be proportional to its mass. The cell species were allowed to move across the lattice by diffusion. The parameters of the system are given in Table 1.

**Table 1:** Parameters used for the simulation of community models 1 and 2.

Parameter	Description	Value	Units	Ref.
*υ*_*sp*_	Cell-specific volume	3.07 x 10^3^	mm^3^ g_DW_^-1^	^a^
*υ*_*met*_	Metabolite-specific volume	7.3 x 10^2^	mm^3^ g^-1^	[38]
*M*_*min*,*sp*_	Minimal dry mass for a single cell	0	g_DW_	Assumed
*M*_*max*,*sp*_	Maximal dry mass for a single cell	1.172 x 10^−12^	g_DW_	[37]
*MW*_*protein*_	Protein molecular weight	7.2 x 10^4^	Da	[38]
*v*_*shrinkage*_	Cell shrinkage rate	1.6 x 10^−2^	h^-1^	Assumed
Parameters specific for community model 1:				
$D_{sp}^{o}$	Diffusion of species *S*. *enterica* and *E*. *coli*.	3 x 10^−9^	mm^2^ ms^-1^	[19]
$D_{met}^{o}$	Diffusion of lactose, O_2_, methionine, and acetate in non-crowded medium.	5 x 10^−6^	mm^2^ ms^-1^	[19]
*V*_*M*,*met*_	Maximum uptake rate of lactose, O_2_, methionine, and acetate	10	mmol g_DW_^-1^ h^-1^	[19]
*K*_*M*,*met*_	Michaelis constant for of lactose, O_2_, methionine, and acetate	1 x 10^−2^	mM	[19]
Parameters specific for community model 2:				
$D_{glucose}^{o}$	Glucose diffusion in water	6.7 x 10^−7^	mm^2^ ms^-1^	[39]
$D_{oxygen}^{o}$	Oxygen diffusion in water	2 x 10^−6^	mm^2^ ms^-1^	[39]
$D_{acetate}^{o}$	Acetate diffusion in water	1.21 x 10^−6^	mm^2^ ms^-1^	[39]
*V*_*M*,*glucose*_	Maximum glucose uptake rate	10	mmol g_DW_^-1^ h^-1^	[40]
*K*_*M*,*glucose*_	Michaelis constant for glucose	1.5 x 10^−2^	mM	[40]
*V*_*M*,*oxygen*_	Maximum oxygen uptake rate	15	mmol g_DW_^-1^ h^-1^	[40]
*V*_*M*,*acetate*_	Maximum acetate uptake rate	17	mmol g_DW_^-1^ h^-1^	[41]
*MW*_*EPS*_	EPS molecular weight in biofilm matrix	2.5 x 10^8^	Da	^b^
*υ*_*EPS*_	Polysaccharide-specific volume	9.2 x 10^3^	mm^3^ g^-1^	^c^

^a^ Computed as *υ*_*sp*_ = *ρ*_*sp*_^-1^ × *M*_*cell*,*wet*_ / *M*_*cell*,*dry*_, where *ρ*_*sp*_ = 1.105 g mL [42], *M*_*cell*,*dry*_ = 2.8 x 10^−13^ g_DW_, and *M*_*cell*,*wet*_ = 9.5 x 10^−13^ g [43].

^b^ Assumed based to be similar to the DNA molecular weight [44].

^c^ Assumed based on [3].

Lactose and O_2_ were supplied to the system to maintain a constant effective concentration of 2.92 mM and 0.21 mM in all boxes, respectively. Other metabolites such as galactose, acetate, and methionine were available in the system only when they were synthesized by microbial species. All metabolites are allowed to escape from the system through the four boundaries, where the metabolite concentrations were set equal to zero. Zero diffusive flux boundaries were applied for bacterial species. Well-mixed conditions were assumed inside each CN-box and IbM-box. All simulations were replicated 5 times. The diffusion of the metabolites were computed using the CN method.

GEM models for the methionine-secreting *S*. *enterica* and *E*. *coli* Δ*metB* were constructed as described by Harcombe et al. [19]. For the *E*. *coli* iJ01366 *core* [45], the reaction catalyzed by the cystathionine-γ-synthase was blocked in the GEM model to prevent the synthesis of methionine. For *S*. *enterica* iRR1083 [46], the biomass reaction was modified to *consume* 0.5 mmol g_DW_^-1^ of intracellular methionine balanced by the *production* of the same amount of methionine that will be secreted to the medium. Furthermore, to simulate the metabolic variability of *S*. *enterica* (see below), the ratio of methionine: biomass (*r*_*meth*_) in the biomass reaction was constrained either to 0.5 or 0 mmol g_DW_^-1^. In this way, different subpopulations were characterized by the methionine production, where a cell with *r*_*meth*_ = 0 corresponds to *S*. *enterica* wild type (WT) that does not secrete methionine (identified as meth^-^), while *r*_*meth*_ = 0.5 corresponds to methionine-secreting *S*. *enterica* mutant (meth^+^).

In an attempt to reduce the computational burden of the IbM simulations, CROMICS approximates the metabolic activity of the metabacteria using NNs. For this purpose, one NN of 2 hidden layers and 15 neurons was created for each species using *v*_*bio*_ and *v*_*f*,*ex*,*met*_ computed by TFA for 30,000 flux samples (see S1 Text Neural Network as an alternative for the computation of metabolic fluxes). For *E*. *coli*, the NN inputs were the upper flux limits *v*^*U*^_*f*_ for lactose, galactose, acetate, and O_2_. For simplicity, only one NN was trained for all *S*. *enterica* subpopulations. For this purpose, the training data were computed by randomly modifying *r*_*meth*_ to either to 0.5 or 0. Thus, the estimation of the *S*. *enterica* metabolism (for both TFA and NN) requires as inputs *v*^*U*^_*f*_ for acetate, galactose, O_2_, and also *r*_*meth*_. The Pearson correlation coefficient and normalized mean squared error (*nmse*) were estimated as 0.9982 and 6.4 x 10^−3^ for *E*. *coli*, and 1 and 4.47 x 10^−4^ for *S*. *enterica*, respectively (S2 and S3 Figs).

NNs significantly reduce the runtime required for metabolic flux estimations, e.g. the exchange fluxes of 10,000 bacteria are computed in approximately 0.04 s (based on the GEM model of *E*. *coli*) using Matlab in a 12-core Intel Xeon E5, CPU 2.7 GHz. Comparatively, the runtime required by TFA for the parallel computation of metabolic distributions (by maximizing the growth rate *v*_*bio*_, using CPLEX) of a similar number of bacteria is about 93 min, and with reduced GEM models [47], the time required is 32 min. Thus, NNs reduce the computational cost associated with the cellular metabolic response in spatio-temporal simulations that require a fine time discretization Δ*t*, with a large number of cells and/or when the metabolic model used is computationally expensive (e.g. genome-scale models of metabolism and macromolecular expression). However, training the NNs requires previous knowledge of the prevalent metabolite exchanged among the species to select the most important metabolites to track. The use of TFA or other stochiometric-based models could be more appropriate in more complex problems, such as when the metabolic flux distributions of a species are very sensitive to small amounts of multiple substrates.

As a second case study, we simulated the co-growth of *E*. *coli* Δ*metB* and two *S*. *enterica* subpopulations (with species ratio 50:50). Twenty bacterial spots were randomly inoculated in the system with similar dimensions as described before. Each spot represents 200 metabacteria of *E*. *coli* and 200 of meth^-^. Only one spot (colony A) contains meth^+^ metabacteria. Three different initial number of meth^+^ metabacteria were tested: 70, 60, and 50 metabacteria. All metabacteria were randomly allocated in the spots.

#### Community model 2: EPS-secreting mutants of *E*. *coli*

The growth of two *E*. *coli* biofilms (identified as Beps^+^ and Beps^++^) on glucose and aerobic conditions were simulated in a 3D system. Biofilm Beps^+^ was composed by (non-EPS-secreting) wild type cells and mutants identified as eps^+^ that secreted 0.11 g g_DW_^-1^ of EPS, while biofilm Beps^++^ contained WT and mutants identified as eps^++^ that secreted 0.43 g g_DW_^-1^ of EPS. We assumed the same metabolic cost for the synthesis of 1 g of EPS than for 1 g_DW_ of biomass.

The CN-lattice was defined by 11 x 11 x 34 cubic boxes (*V*_*CN-box*_ = 1.4 x 10^−6^ mm^3^), while the IbM-lattice was divided into 22 x 22 x 68 cubic boxes of Δ*x* = 5.7 x 10^−3^ mm per side (i.e., *V*_*IbM-box*_ = 1.8 x 10^−7^ mm^3^). Each CN-box contains 8 IbM-boxes, and 1 IbM-box can allocate at most one metabacterium. To take into account that eps^+^ and eps^++^ accumulated EPS, a therefore a lower number of cells can be allocated in IbM-box, the number of cells per metabacterium was computed as *metaB*_*sp*_ = 0.52Δ*x*_*IbM*_Δ*y*_*IbM*_Δ*z*_*IbM*_/(*M*_*max*,*sp*_*υ*_*sp*_+*M*_*EPS*,*sp*_*υ*_*EPS*_), where the denominator represents the maximum volume occupied by a cell of species *sp* and the EPS secreted by this. We assumed that the EPS amount *M*_*EPS*,*sp*_ present in a box is proportional to the cell mass, thus $M_{EPS,{eps}^{+}}={0.11M}_{max,{eps}^{+}}$ and $M_{EPS,{eps}^{++}}={0.43M}_{max,{eps}^{++}}$ for eps mutants, while *M*_*EPS*,*WT*_ = 0 for the non-EPS-secreting WT. Thus, one metabacterim represents 27 cells of WT, 20 of eps^+^, and 12 of eps^++^. The volume occupied in a box by the cells and EPS were similar for the three species. The maximal metabacterium mass (or mass threshold to carry out the cell division) was then obtained by multiplying *M*_*max*,*sp*_ of a single cells (Table 1) by *metaB*_*sp*_.

In both biofilms Beps^+^ and Beps^++^, the system is initialized with 1,220 metabacteria randomly allocated at the bottom of the system, the left side of the biofilm is composed by 610 *E*. *coli* WT cells, while an equal number eps mutant cells are located on the right side. As in the previous community model, the initial mass of each metabacterium was randomly taken from the same normal distribution. We assumed that the cells were attached to a biofilm and that their motion is only due to the cell shoving, i.e., *D*_*sp*_ = 0. Δ*t* = Δ*t*_*CN*_ = 10.83 ms.

Periodic boundary conditions were assumed in the *x* and *y* directions for both metabolites and bacterial species, while a zero diffusive flux was located at the bottom of the system. The nutrients glucose and O_2_ were supplied from the top of the boundary, where the concentrations of 5 mM of glucose and 0.21 mM of O_2_ were kept constant. Neither the metabolites nor cells were allowed to leave the system through the top boundary. All CN-boxes contained an initial amount of nutrients equivalent to 5 mM of glucose and 0.21 mM of O_2._ The diffusion of the metabolites was computed using the cLBM approach.

All molecules (metabolites, EPS, and cells) are assumed to be spherical shape and with a volume proportional to their molecular weight (see S1 Text Eq S3). Only the volume of macromolecules with a molecular weight greater than 400 Da were explicitly considered in the simulations. All simulations were replicated 5 times.

As in the previous case study, GEM models for the eps mutants can be constructed from *E*. *coli* WT iJ01366 [47] by modifying the biomass reaction to produce either 0.11 g g_DW_^-1^ or 0.43 g g_DW_^-1^ of EPS that will be secreted to the medium by eps^+^ and eps^++^, respectively. NNs of 2 hidden layers with 15 neurons each were created using 30,000 flux solutions computed by TFA for the GEM models. The inputs for the NNs were the upper flux limits *v*^*U*^_*f*_ for glucose, acetate, and O_2_. The Pearson correlation coefficient was estimated as 1, and *nmse* as 2.4 x 10^−5^ (S4 Fig).

### Calculating the species fitness in the community models

The average fitness or growth of a species *sp* at the end of the simulation was computed as

$${Fitness}_{sp}=\frac{1}{t_{sim}}ln\left(\frac{\rho_{sp}(t_{sim})}{\rho_{sp}(0)}\right)$$ (10)

where *ρ*_*sp*_(*t*) is the total biomass of species *sp* at *t*, and *t*_*sim*_ is the final simulation time. Additionally, the relative fitness of species *sp*1 in competition with *sp*2 can be computed as

$$w_{sp1}={Fitness}_{sp1}/{Fitness}_{sp2}$$ (11)

## Supporting information

S1 Fig *E*. *coli* Δ*metB* and methionine-secreting mutant of *S*. *enterica* consortium. (A) Schematic representation of the microbial community in a 2D system. (B, C) The species ratio convergence predicted by CROMICS, COMETS, and the experimental observations [19] after 48 h for an initial composition *E*. *coli*: *S*. *enterica* of (B) 99:1 and (C) 1:99.
(TIF)

S2 Fig Parity and residual plots of the metabolic fluxes estimated by neural networks (NN) and thermodynamics flux analysis (TFA) for *E*. *coli* Δ*metB*. To train a NN with 2 layers of 15 neurons each, 30,000 flux samples were used. Training data were obtained by assuming that for a given uptake flux of lactose, O_2_, and methionine, the cells produce a mean flux value of acetate, galactose, and growth rate. The Pearson correlation r was estimated as 0.9982, while the normalized mean square error between the fluxes predicted by TFA and NN was estimated to be 6.4 x 10^−3^. Fluxes *v*_*f*_ are given in mmol g_DW_^-1^ h^-1^, and *v*_*bio*_ in h^-1^.
(TIF)

S3 Fig Parity and residual plots of the metabolic fluxes estimated by NN and TFA for *S*. *enterica*. To train a NN with 2 layers of 15 neurons each, 30,000 flux samples were used. Training data were obtained by assuming that for a given uptake flux of acetate, galactose, O_2_, and methionine:biomass ratio *r*_*meth*_, the cells produce a mean flux value of methionine and growth rate. The Pearson correlation r was estimated as 1, while the normalized mean square error between the fluxes predicted by TFA and NN was estimated to be 4.47 x 10^−4^. Fluxes *v*_*f*_ are given in mmol g_DW_^-1^ h^-1^, and *v*_*bio*_ in h^-1^.
(TIF)

S4 FigParity and residual plots of the metabolic fluxes estimated by NN and TFA for *E*. *coli WT*.To train a NN with 2 layers of 15 neurons each, 30,000 flux samples were used. Training data were obtained by assuming that for a given uptake flux of glucose and O_2_, the cells produce a mean flux value of acetate and growth rate. The Pearson correlation r was estimated as 1, while the normalized mean square error between the fluxes predicted by TFA and NN was estimated to be 2.4 x 10^−5^. Fluxes *v*_*f*_ are given in mmol g_DW_^-1^ h^-1^, and *v*_*bio*_ in h^-1^. GEM models for the eps^+^ and eps^++^ mutants were constructed by modifying the biomass reaction to produce 0.11 g g_DW_^-1^ and 0.43 g g_DW_^-1^ of EPS that will be secreted to the medium. In comparison when the same metabolic upper flux limits were used, the growth rate computed by TFA for mutants were $v_{bio,{eps}^{+}}=0.9v_{bio,WT}$, and $v_{bio,{eps}^{++}}=0.7v_{bio,WT}$, while the other metabolic fluxes (glucose, O_2_, and acetate) predicted were the same for the three type *E*. *coli*. Thus, for simplicity, the NN created for WT was modified to represent the eps^+^ and eps^++^, by multiplying the biomass computed by the original NN_WT_ by a factor of 0.9 and 0.7, respectively.(TIF)

S1 Text Considerations and methodologies used in CROMICS. (DOCX)

## List of abbreviations

Beps^+^: Multispecies biofilm composed by *E*. *coli* wild type and eps^+^ mutans
Beps^++^: Multispecies biofilm composed by *E*. *coli* wild type and eps^++^ mutans
cLBM: Crowding adaptation of the lattice Bolzmann method
CN: Crank-Nicholson method
CROMICS: Crowding-modeling of in-silico community systems
eps^+^: *E*. *coli* mutant that secretes 0.11 g gDW-1 of EPS
eps^++^: *E*. *coli* mutant that secretes 0.43 g gDW-1 of EPS
EPS: Extracellular polymeric substances
GEM: Genome-scale metabolic models
IbM: Individual-based modeling
nmse: Normalized mean squared error
NN: Neural network
SPT: Scaled particle theory
TFA: Thermodynamic flux analysis
WT: Wild type cell
