# Supplementary figures and images for: Spatio-temporal modeling of the crowding conditions and metabolic variability in microbial communities
Source: PLoS Comput Biol. 2021 Jul 22;17(7):e1009140. doi: 10.1371/journal.pcbi.1009140 (PMC8297787; doi:10.1371/journal.pcbi.1009140)

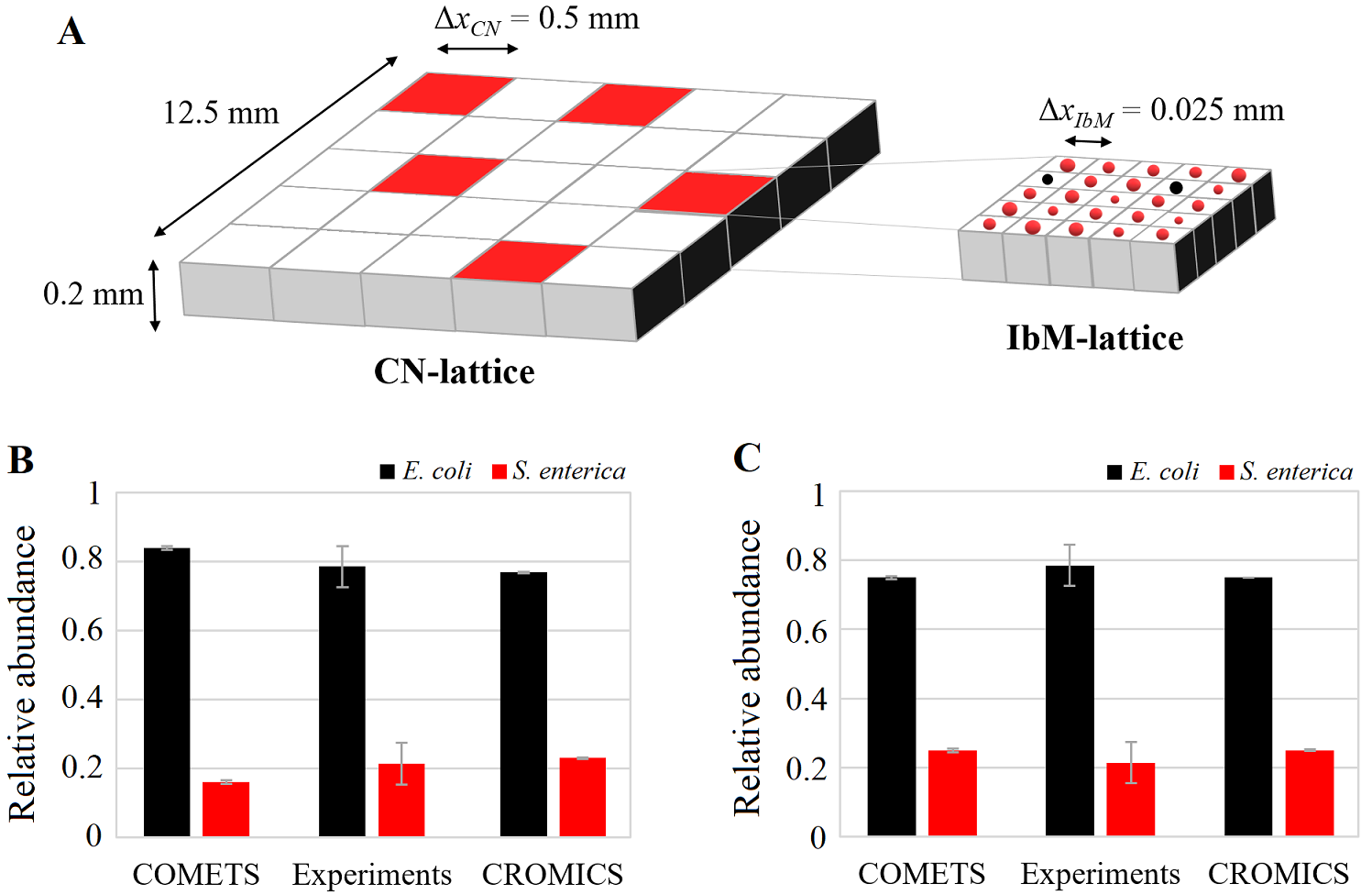

Supplement: S1 Fig — (A) Schematic representation of the microbial community in a 2D system. (B, C) The species ratio convergence predicted by CROMICS, COMETS, and the experimental observations [19] after 48 h for an initial composition E. coli: S. enterica of (B) 99:1 and (C) 1:99. (TIF) [file pcbi.1009140.s001.tif]

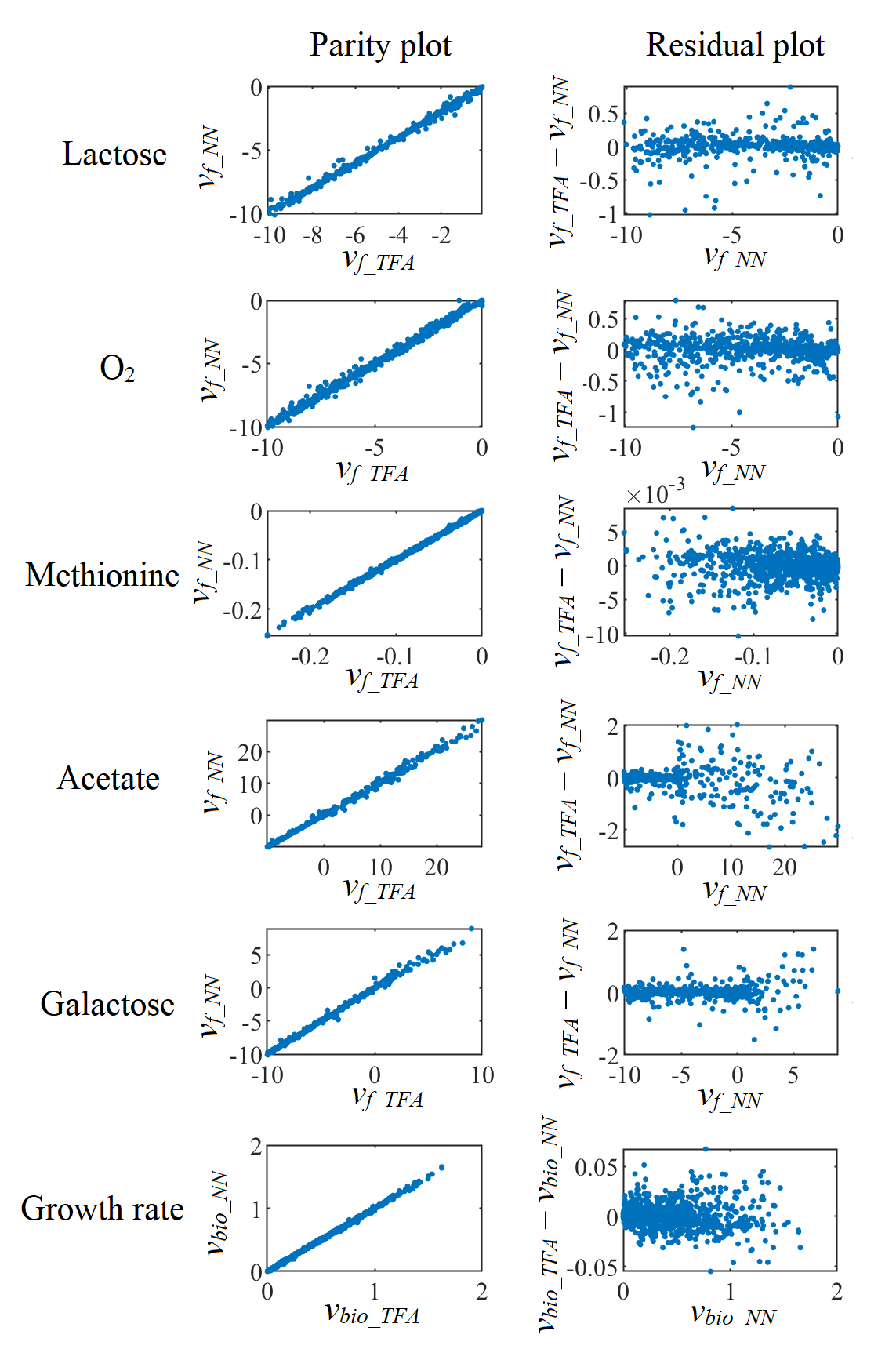

Supplement: S2 Fig — To train a NN with 2 layers of 15 neurons each, 30,000 flux samples were used. Training data were obtained by assuming that for a given uptake flux of lactose, O2, and methionine, the cells produce a mean flux value of acetate, galactose, and growth rate. The Pearson correlation r was estimated as 0.9982, while the normalized mean square error between the fluxes predicted by TFA and NN was estimated to be 6.4 x 10^−3^. Fluxes vf are given in mmol gDW-1 h-1, and vbio in h-1. (TIF) [file pcbi.1009140.s002.tif]

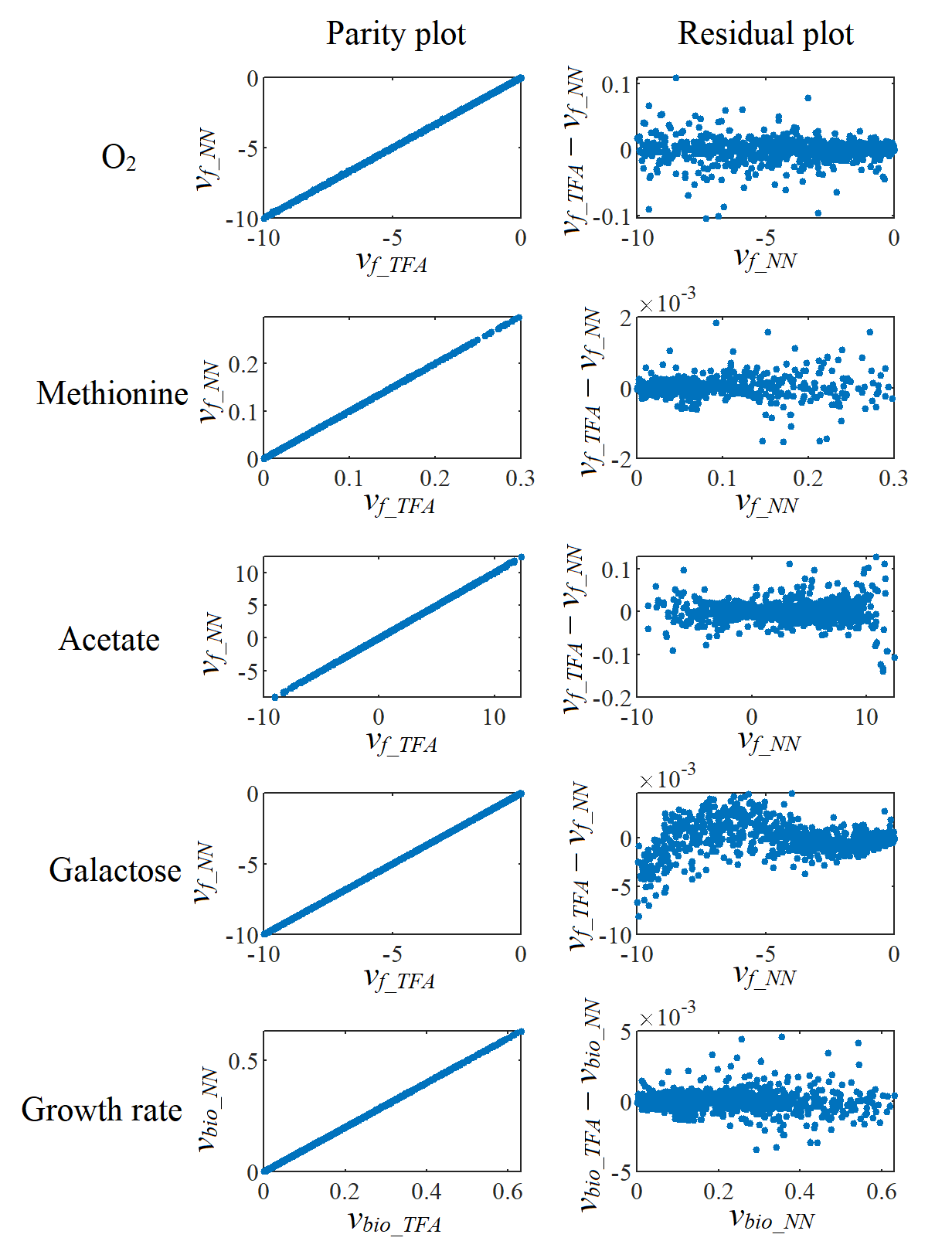

Supplement: S3 Fig — To train a NN with 2 layers of 15 neurons each, 30,000 flux samples were used. Training data were obtained by assuming that for a given uptake flux of acetate, galactose, O2, and methionine:biomass ratio rmeth, the cells produce a mean flux value of methionine and growth rate. The Pearson correlation r was estimated as 1, while the normalized mean square error between the fluxes predicted by TFA and NN was estimated to be 4.47 x 10^−4^. Fluxes vf are given in mmol gDW-1 h-1, and vbio in h-1. (TIF) [file pcbi.1009140.s003.tif]

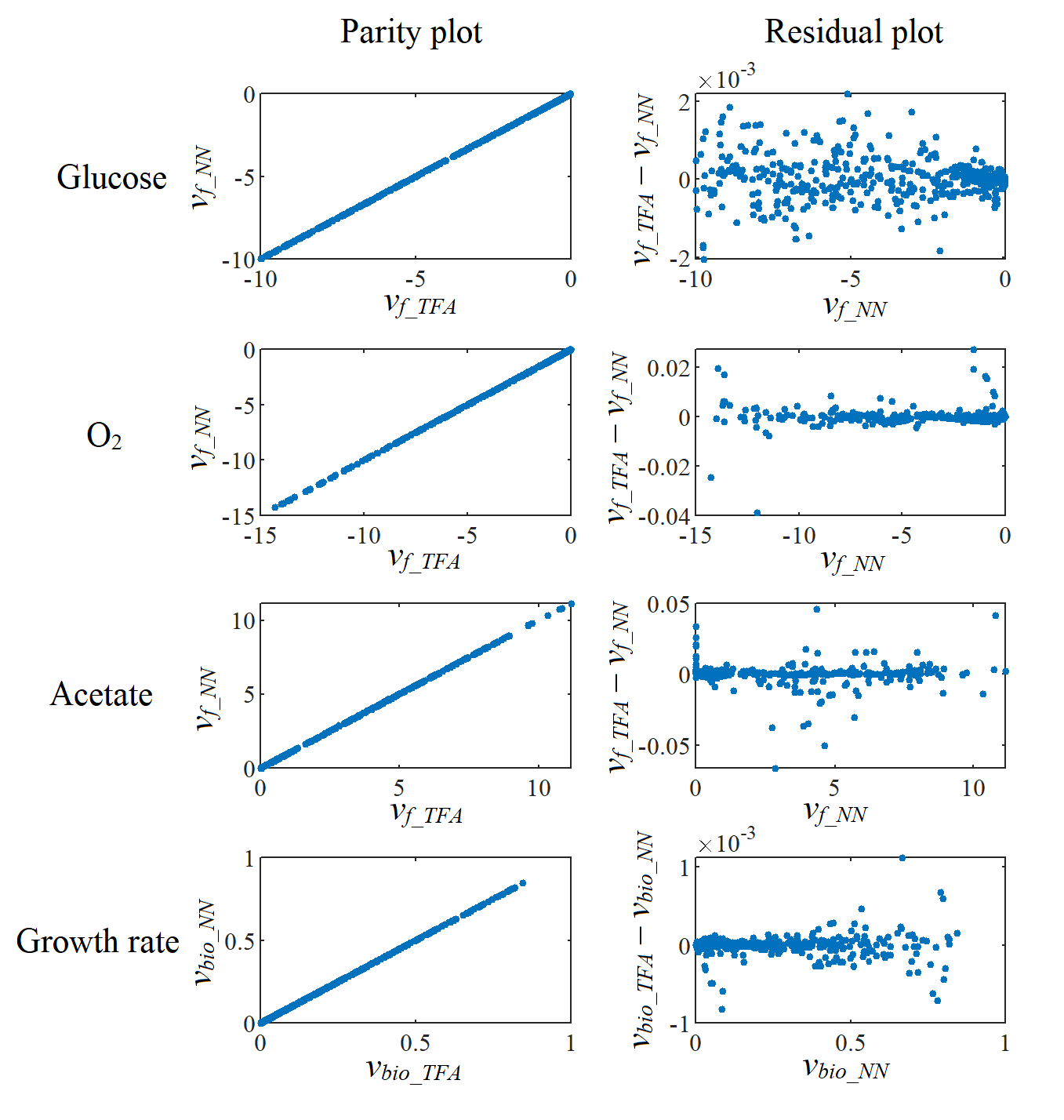

Supplement: S4 Fig — To train a NN with 2 layers of 15 neurons each, 30,000 flux samples were used. Training data were obtained by assuming that for a given uptake flux of glucose and O2, the cells produce a mean flux value of acetate and growth rate. The Pearson correlation r was estimated as 1, while the normalized mean square error between the fluxes predicted by TFA and NN was estimated to be 2.4 x 10^−5^. Fluxes vf are given in mmol gDW-1 h-1, and vbio in h-1. GEM models for the eps+ and eps++ mutants were constructed by modifying the biomass reaction to produce 0.11 g gDW-1 and 0.43 g gDW-1 of EPS that will be secreted to the medium. In comparison when the same metabolic upper flux limits were used, the growth rate computed by TFA for mutants were vbio,eps+=0.9vbio,WT, and vbio,eps++=0.7vbio,WT, while the other metabolic fluxes (glucose, O2, and acetate) predicted were the same for the three type E. coli. Thus, for simplicity, the NN created for WT was modified to represent the eps+ and eps++, by multiplying the biomass computed by the original NNWT by a factor of 0.9 and 0.7, respectively. (TIF) [file pcbi.1009140.s004.tif]
