# Supplementary material for: Spatio-temporal modeling of the crowding conditions and metabolic variability in microbial communities
Source: PLoS Comput Biol. 2021 Jul 22;17(7):e1009140. doi: 10.1371/journal.pcbi.1009140 (PMC8297787; doi:10.1371/journal.pcbi.1009140)
Supplement: S1 Text — (DOCX) [file pcbi.1009140.s005.docx]

# S1 Text

# Spatio-temporal modeling of the crowding conditions and metabolic variability in microbial communities

Liliana Angeles-Martinez, Vassily Hatzimanikatis

# **Coarse-grained considerations**

The metabolite diffusion process and the distribution of microbial species are simulated on two different lattices identified as IbM (for individual-based model) and Crank-Nicholson (CN) lattice, respectively. Therefore, the substrate availability for each cell as well as the free space information are determined by the superimposition of both lattices. In the IbM lattice, the box volume Δ*x_IbM_^3^* is determined by the maximum size that a cell can reach before cellular division occurs. However, a coarse-grained discretization Δ*x_CN_* can be applied for the diffusion simulation of the metabolites, i.e., Δ*x_CN_* > Δ*x_IbM_*, where several cells share the same substrate concentration contained in a box *ijk* with volume $V_{box}={\Delta x}_{CN}{\Delta y}_{CN}{\Delta z}_{CN}$*.*

If two or more cells share the same box *ijk*, the maximum uptake rate $v_{f,ex,met}^{U,s}$ for each cell *s* in the box is given by

$v_{f,ex,met}^{U,s}=\min\left( v_{f,ex,met}^{U},\frac{\rho_{met}(ijk,t)}{\sum_{s}^{Ncells(ijk)} v_{f,ex,met}^{U}M_{cell,s}(t)\Delta t}v_{f,ex,met}^{U} \right)$. (S1)

Eq. S1 guarantees that the total amount of *met* consumed in the box ($\sum_{s}^{Ncells(ijk)} v_{f,ex,met}^{U}M_{cell,s}\Delta t$) does not exceed the amount available (*ρ_met_*). Thus, when *ρ_met_* is in excess and $\rho_{met}/\sum_{s}^{Ncells\left( ijk \right)} v_{f,ex,met}^{U}M_{cell,s}\Delta t>1$, then $v_{f,ex,met}^{U,s}$ of each cell *s* is equal to *v^U^_f,ex,met_* estimated by the Michaelis-Menten equation (Eq. 1 of the main text). Conversely, when $\rho_{met}/\sum_{s}^{Ncells\left( ijk \right)} v_{f,ex,met}^{U}M_{cell,s}\Delta t<1$, then the available *met* is split among the cells so that *v^U^_f,ex,met_* is weighted by the fraction availability/consumption of metabolite *met* to (re)calculate the maximum uptake rate $v_{f,ex,met}^{U,s}$ per cell *s* in box *ijk*.

# **SPT considerations**

Even though some metabolites such as O_2_ are able to penetrate the cell membrane, the presence of intracellular macromolecules (DNA, ribosomes, proteins, etc.) reduce the available volume [1]. In these cases, *γ_met_* (Eq. 3 and 4 of the meain text) is no longer a function of *R_cell_* but of the radii *R_protein_* and the abundance of the intracellular proteins of 72 kDa [2]. Assuming that the total mass of intracellular proteins is equivalent to the dry cell mass *M_cell_*, then Eq. 4 of the main text becomes

$S_{x}=\frac{\pi}{6V_{box}}\left( \sum_{l}^{macromolecules} \frac{\rho_{l}N_{A}}{{10}^{3}}\left( 2R_{l} \right)^{x}+\sum_{l}^{cells} {\frac{M_{cell}N_{A}}{{MW}_{protein}}\left( 2R_{protein} \right)}^{x} \right). x=1,2,3$ (S2)

The term $M_{cell}N_{A}/{MW}_{protein}$ represents the number of proteins inside each cell, where *MW* is the molecular weight. Finally, assuming a constant specific volume *υ_met_*, the radii *R_met_* and *R_protein_* can be estimated as

$R_{met}=\left( 3{MW}_{met}\upsilon_{met}/4\pi N_{A} \right)^{1/3}.$ (S3)

# **Metabolic flux estimations**

In CROMICS, each microbial cell is represented by an agent that interacts with the environment and other neighboring agents. The interactions between a cell and the local environment are determined by the metabolic capabilities of the microorganism, as in its ability to grow on different types of substrates, the uptake/production rates of metabolites, etc. Stoichiometric-based models (see [3] for a review) have been widely used for estimating the growth rate and the metabolic flux distributions in an organism. In this paper, we use thermodynamics flux analysis (TFA) [4], though other stoichiometric models and constraints can also be applied.

TFA is a mixed*-*integer linear problem (MILP) seeking to maximize the growth rate *ν_bio_* [h^-1^], subject to mass conservation and thermodynamics [4].

$\max v_{bio}$ (S4a)

$s.t. \mathbf{N}\cdot\mathbf{v}$ (S4b)

$0\leq v_{f}\leq{z_{f}v}_{f}^{U}$ (S4c)

$\Delta_{r}{G'}_{f}-Q+Qz_{f}<0$ (S4d)

$\Delta_{r}{G'}_{f}-RT\sum_{a=1}^{m} \eta_{f,a}c_{a}-\Delta_{r}G_{f}^{'o}<0$ (S4e)

$c_{a}^{L}\leq c_{a}\leq c_{a}^{U}$ (S4f)

The flux vector **v** [*n* x 1] contains the (irreversible) forward and backward components of each reaction flux, while the matrix **N** [*m* x *n*] contains the stoichiometric information of *m* intracellular metabolites and *n* intracellular and transport reactions. Since all reaction fluxes have been split into its forward and backward components, *v_f_* can take any value between 0 and an upper limit *v^U^_f_* (Eq. S4c). Here, the binary variable *z_f_* is equal to 1 when *v_f_* is positive, while *z_f_* is 0 when *v_f_* is 0. *Q* is large number constant (e.g. *Q* = 5,000). Eq. S4d guarantees that *z_f_* is equal to 1 only if the Gibbs free energy of reaction *f*, ${\text{∆}_{\text{r}}\text{G}}_{\text{f}}^{'}$, is negative. Therefore, through Eq. S4c and S4d, the flux of reaction *f* is allowed only in the direction of the Gibbs energy drop. ${\text{∆}_{\text{r}}\text{G}}_{\text{f}}^{'}$ (Eq. S4e) is a function of the standard Gibbs free energy at physiological conditions (${\text{∆}_{\text{r}}\text{G}}_{\text{f}}^{'o}$) and the logarithm of the intracellular concentration of metabolite *a*, $\text{c}_{\text{a}}\text{ = ln(}\text{C}_{\text{int,a}}\text{/}\text{C}_{\text{0}}\text{)}$. Here, the intracellular concentration $\text{C}_{\text{int,a}}$ is normalized by the standard concentration *C_0_* = 1 M. The value of ${\text{∆}_{\text{r}}\text{G}}_{\text{f}}^{'o}$ is estimated using the group-contribution method [5]. *R* represents the ideal gas constant, *T* is the temperature, and $\text{η}_{\text{f,a}}$ is the stoichiometric coefficient of metabolite *a* in reaction *f*. The typical value of 100 mmol g_DW_^-1^ h^-1^ was chosen for the upper flux limit *v^U^_f_*, while the concentration limits were set to *C_a,min_* = 10^-6^ M and *C_a,max_* = 0.05 M [4]. The uptake flux limits *v^U^_f,ex,met_* are computed based on the effective nutrient concentration in the medium (Eq. 1 and 2 of the main text).

The optimal solutions for *ν_bio_* and *ν_f,ex,met_* of the metabolites consumed/produced were computed using the CPLEX solver. Then, *ν_bio_* and *ν_f,ex,met_* were used to update the cell mass *M_cell_* and the amount metabolite *ρ_met_* in each box for the next time *t* + Δ*t* (Eq. 5 and 6 of the main text).

## **Neural Network as an alternative for the computation of metabolic fluxes**

The simulation of large microbial systems involves a large number of TFA evaluations to update *M_cell_*, *R_cell_*, and *ρ_met_* at every Δ*t_CN_*. For example, when simulations with fine space/time discretization are required, Δ*t_CN_* can grow to the order of 10^-3^ s, so to simulate 1 h of growth of 10^3^ individual cells, we would need to solve ~10^9^ TFA optimization problems. To reduce the computation burden associated with the calculation of metabolic fluxes, we approximate the TFA solutions (*v_f,ex,met_* and *v_bio_*) using Neural Networks (NNs) [6]. One NN is created for each microbial species or GEM model. Similarly to any stoichiometric-based models, NN predicts flux outputs such as *ν_bio_* and *ν_f,ex,met_* from the input data given by the uptake flux limits *v^U^_f,ex,met_*. The procedure to create an NN can be summarized as follows:

1. Sample generation. We randomly sample different combinations of uptake flux values *v^U^_f,ex,met_* (between 0 and *V_M,met_*) for all the main substrates present in the system, e.g. lactose, methionine, O_2_, and acetate. In this paper, we generate 30,000 flux samples for each microbial species.
2. Compute the training data. The samples generated before are used to constrain the optimization problem posed by TFA. The exchange fluxes of minor metabolite components in the metabolic network were left unconstrained, i.e., *v^U^_f,ex,met_* = 1,000 mmol g_DW_^-1^ h^-1^. To reduce the multiplicity in the metabolic flux distributions and find a representative flux distribution, two consecutive TFA optimizations (Eq. S4) were performed with different objective functions: (i) maximization of *v_bio_*, (ii) minimization of the sum of the fluxes. The value *v_bio_* found in the first optimization is used as constraint in the subsequent minimization of $\sum_{f} v_{f}$.
3. NN training. Deep learning toolbox of Matlab R2018b was used to train the NN. For this purpose, we set the *v^U^_f,ex,met_* samples generated in step 1 as inputs, while the flux solutions *v_bio_* and *v_f,ex,met_* computed by TFA were set as outputs.

# **Metabolite diffusion**

## **Crank-Nicholson approach**

The diffusion of metabolites in a 3D system can be computed by solving the diffusion equation $\frac{\partial\rho_{met}}{\delta t}=\nabla\cdot(D_{eff,met}{\nabla\rho}_{met})$, where the effective diffusion coefficient *D_eff_* is a function of the available space at time *t*, and *ρ_met_* is the amount of metabolites [mmol] per box, i.e., *ρ_met_* = *C_met_* *V_box_*.

We use the fractional steps method (FSM) and a semi-implicit Crank-Nicholson (CN) approach [7,8] to solve the 3-dimensional partial differential equation (PDE) posed by the diffusion equation. In the FSM, the diffusion equation is split into its spatial coordinates [7]:

$\frac{1}{3}\frac{\partial\rho_{met}}{\delta t}=\frac{\partial^{2}(D_{eff,met}\rho_{met})}{\delta x^{2}},$ (S5)

$\frac{1}{3}\frac{\partial\rho_{met}}{\delta t}=\frac{\partial^{2}(D_{eff,met}\rho_{met})}{\delta y^{2}}$, (S6)

$\frac{1}{3}\frac{\partial\rho_{met}}{\delta t}=\frac{\partial^{2}(D_{eff,met}\rho_{met})}{\delta z^{2}}$. (S7)

Eq. S5–S7 are solved consecutively to integrate the amount of metabolite *ρ_met_* over the entire Δ*t_CN_*. Thus, the solution of the 3D diffusion equation is found by solving the three one-dimensional PDEs.

To incorporate the crowding effect on the diffusion simulation, we set the first spatial derivative of the *ρ_met_* as the flux of metabolite *met*, *J_met_*_(_*_i,j,k🡪i+_*_1_*_,j,k_*_)_, from box (*i*+1,*j,k*) to (*i*,*j*,*k*) under crowding conditions [9]:

$J_{met\left( i,j,k\to i+1,j,k \right)}=\frac{\partial(D_{eff,met}\rho_{met})}{\delta x}=-\frac{D_{met}^{o}}{V_{box}\Delta x}\left( P_{met\left( i,j,k \right)}\rho_{met\left( i+1,j,k \right)}-P_{met\left( i+1,j,k \right)}\rho_{met\left( i,j,k \right)} \right).$ (S8)

In Eq. S8, the effective diffusion in each box is given by $D_{eff,met}=D_{met}^{o}P_{met(i,j,k)}$ [10], where $D_{met}^{o}$ is the diffusion coefficient in diluted solutions. The probability *P_met_* of metabolite *met* finding available space in box (*i,j,k*) is equal 1/*γ_met_*. Scaled particle theory (SPT) is used to estimate the activity coefficient *γ_met_* (Eq. 3 of the main text).

After applying Eq. S5 to find the second spatial derivative in the CN method, the one-dimensional diffusion equation in the *x-*direction (Eq. S5) becomes

$\frac{\rho_{met(i,j,k)}^{t+\Delta t_{CN}/3}-\rho_{met(i,j,k)}^{t}}{\Delta t_{CN}}=-\frac{D_{met}^{o}}{2}\left( \frac{P_{met\left( i,j,k \right)}^{t+\frac{{\Delta t}_{CN}}{3}}\rho_{met\left( i-1,j,k \right)}^{t+\frac{\Delta t_{CN}}{3}}-\left( P_{met\left( i-1,j,k \right)}^{t+\frac{{\Delta t}_{CN}}{3}}+P_{met\left( i+1,j,k \right)}^{t+\frac{{\Delta t}_{CN}}{3}} \right)\rho_{met\left( i,j,k \right)}^{t+\frac{\Delta t_{CN}}{3}}+P_{met\left( i,j,k \right)}^{t+\frac{\Delta t_{CN}}{3}}\rho_{met\left( i+1,j,k \right)}^{t+\frac{{\Delta t}_{CN}}{3}}}{\Delta x^{2}}+\frac{P_{met\left( i,j,k \right)}^{t}\rho_{met\left( i-1,j,k \right)}^{t}-\left( P_{met\left( i-1,j,k \right)}^{t}+P_{met\left( i+1,j,k \right)}^{t} \right)\rho_{met\left( i,j,k \right)}^{t}+P_{met\left( i,j,k \right)}^{t}\rho_{met\left( i+1,j,k \right)}^{t}}{\Delta x^{2}} \right).$ (S9)

In the *y-*direction, Eq. S6 becomes

$\frac{\rho_{met(i,j,k)}^{t+2{\Delta t}_{CN}/3}-\rho_{met(i,j,k)}^{t+\Delta t_{CN}/3}}{\Delta t_{CN}}=-\frac{D_{met}^{o}}{2}\left( \frac{P_{met(i,j,k)}^{t+{2\Delta t}_{CN}/3}\rho_{met\left( i,j-1,k \right)}^{t+2\Delta t_{CN}/3}-\left( P_{met\left( i,j-1,k \right)}^{t+{2\Delta t}_{CN}/3}+P_{met\left( i,j+1,k \right)}^{t+2\Delta t_{CN}/3} \right)\rho_{met\left( i,j,k \right)}^{t+{2\Delta t}_{CN}/3}+P_{met(i,j,k)}^{t+{2\Delta t}_{CN}/3}\rho_{met\left( i,j+1,k \right)}^{t+2\Delta t_{CN}/3}}{\Delta y^{2}}+\frac{P_{met(i,j,k)}^{t+\Delta t_{CN}/3}\rho_{met\left( i,j-1,k \right)}^{t+\Delta t_{CN}/3}-\left( P_{met\left( i,j-1,k \right)}^{t+{\Delta t}_{CN}/3}+P_{met\left( i,j+1,k \right)}^{t+{\Delta t}_{CN}/3} \right)\rho_{met\left( i,j,k \right)}^{t+\Delta t_{CN}/3}+P_{met(i,j,k)}^{t+\Delta t_{CN}/3}\rho_{met\left( i,j+1,k \right)}^{t+\Delta t_{CN}/3}}{\Delta y^{2}} \right).$ (S10)

And, in the *z-*direction, Eq. S7 becomes

$\frac{\rho_{met(i,j,k)}^{t+{\Delta t}_{CN}}-\rho_{met(i,j,k)}^{t+\Delta t_{CN}/3}}{\Delta t_{CN}}=-\frac{D_{met}^{o}}{2}\left( \frac{P_{met\left( i,j,k \right)}^{t+{\Delta t}_{CN}}\rho_{met\left( i,j,k-1 \right)}^{t+\Delta t_{CN}}-\left( P_{met\left( i,j,k-1 \right)}^{t+{\Delta t}_{CN}}+P_{met\left( i,j,k+1 \right)}^{t+\Delta t_{CN}} \right)\rho_{met\left( i,j,k \right)}^{t+{\Delta t}_{CN}}+P_{met\left( i,j,k \right)}^{t+{\Delta t}_{CN}}\rho_{met\left( i,j,k+1 \right)}^{t+\Delta t_{CN}}}{\Delta z^{2}}+\frac{P_{met\left( i,j,k \right)}^{t+\frac{2\Delta t_{CN}}{3}}\rho_{met\left( i,j,k-1 \right)}^{t+\frac{2\Delta t_{CN}}{3}}-\left( P_{met\left( i,j,k-1 \right)}^{t+\frac{{2\Delta t}_{CN}}{3}}+P_{met\left( i,j,k+1 \right)}^{t+\frac{{2\Delta t}_{CN}}{3}} \right)\rho_{met\left( i,j,k \right)}^{t+\frac{2\Delta t_{CN}}{3}}+P_{met\left( i,j,k \right)}^{t+\frac{2\Delta t_{CN}}{3}}\rho_{met\left( i,j,k+1 \right)}^{t+\frac{2\Delta t_{CN}}{3}}}{\Delta z^{2}} \right).$ (S11)

The superscripts *t*, *t*+Δ*t_CN_*/3, *t*+2Δ*t_CN_*/3, and *t*+Δ*t_CN_* indicate the time step at which the variable is measured. Assuming that crowding conditions do not change drastically for small Δ*t_CN_* values, then $P_{met\left( i,j,k \right)}^{t}=P_{met\left( i,j,k \right)}^{t+{\Delta t}_{CN}/3}=P_{met\left( i,j,k \right)}^{t+{2\Delta t}_{CN}/3}=P_{met\left( i,j,k \right)}^{t+{\Delta t}_{CN}}$. In this CN scheme, the solution $\rho_{met\left( i,j,k \right)}^{t+\Delta t_{CN}/3}$ computed from *ρ_met_* at a previous time *t* (Eq. S9) is used next to calculate $\rho_{met\left( i,j,k \right)}^{t+2\Delta t_{CN}/3}$ with Eq. S10, and finally, the latter is used to compute the final $\rho_{met\left( i,j,k \right)}^{t+\Delta t_{CN}}$(Eq. S11).

To update *ρ_met_* in the *x*-direction at the new time *t*+Δ*t_CN_*/3 from the data given at *t,* Eq. S9 is rearranged in a matrix form as

$$\left[ \begin{matrix} 1+\lambda_{x}(P_{met\left( 0 \right)}+P_{met\left( 0 \right)}) & {-\lambda}_{x}P_{met\left( 1 \right)} & & & & & \\ {-\lambda}_{x}P_{met\left( 2 \right)} & 1+\lambda_{x}(P_{met\left( 1 \right)}+P_{met\left( 3 \right)}) & {-\lambda}_{x}P_{met\left( 2 \right)} & & & & \\ & & & \ddots& & & \\ & & & & {-\lambda}_{x}P_{met\left( N_{x}-1 \right)} & 1+\lambda_{x}(P_{met\left( N_{x}-2 \right)}+P_{met\left( N_{x} \right)}) & {-\lambda}_{x}P_{met\left( N_{x}-1 \right)} \\ & & & & & {-\lambda}_{x}P_{met\left( N_{x} \right)} & 1+\lambda_{x}(P_{met\left( N_{x}-1 \right)}+P_{met\left( N_{x}+1 \right)}) \end{matrix} \right]\cdot\left[ \begin{matrix} \rho_{met\left( 1 \right)}^{t+\frac{1}{3}} \\ \rho_{met\left( 2 \right)}^{t+\frac{1}{3}} \\ \vdots\\ \rho_{met\left( N_{x}-1 \right)}^{t+\frac{1}{3}} \\ \rho_{met\left( N_{x} \right)}^{t+\frac{1}{3}} \end{matrix} \right]=\left[ \begin{matrix} 1-\lambda_{x}\left( P_{met\left( 0 \right)}+P_{met\left( 0 \right)} \right) & \lambda_{x}P_{met\left( 1 \right)} & & & & & \\ \lambda_{x}P_{met\left( 2 \right)} & 1-\lambda_{x}\left( P_{met\left( 1 \right)}+P_{met\left( 3 \right)} \right) & \lambda_{x}P_{met\left( 2 \right)} & & & & \\ & & & \ddots& & & \\ & & & & \lambda_{x}P_{met\left( N_{x}-1 \right)} & 1-\lambda_{x}\left( P_{met\left( N_{x}-2 \right)}+P_{met\left( N_{x} \right)} \right) & \lambda_{x}P_{met\left( N_{x}-1 \right)} \\ & & & & & \lambda_{x}P_{met\left( N_{x} \right)} & 1-\left( P_{met\left( N_{x}-1 \right)}+P_{met\left( N_{x}+1 \right)} \right) \end{matrix} \right]\cdot\left[ \begin{matrix} \rho_{met\left( 1 \right)}^{t} \\ \rho_{met\left( 2 \right)}^{t} \\ \vdots\\ \rho_{met\left( N_{x}-1 \right)}^{t} \\ \rho_{met\left( N_{x} \right)}^{t} \end{matrix} \right],$$

(S12)

where $\lambda_{x}=\frac{\Delta t_{CN}D_{met}^{o}}{2\Delta x^{2}}$. For boxes located at the boundary of the *x*-axis, e.g. box (1) and (*Nx*) in Eq. (S12), the probability $P_{met(i-1,j,k)}$ and $P_{met(i+1,j,k)}$ can be computed by applying the corresponding boundary condition, e.g. for periodic boundaries, $P_{met\left( 0 \right)}=P_{met(N_{x})}$, or for zero-diffusive flux conditions, $P_{met\left( 0 \right)}=0$. Note that for non-crowded systems, i.e., diluted ones, Eq. S12 resembles the well-known tridiagonal matrix of the CN scheme.

A similar set of matrix equations can be obtained from Eq. S10 and S11 for the corresponding coordinates *y* and *z*, with $\lambda_{y}=\frac{\Delta t_{CN}D_{met}^{o}}{2\Delta y^{2}}$ and $\lambda_{z}=\frac{\Delta t_{CN}D_{met}^{o}}{2\Delta z^{2}}$.

## **cLBM approach**

Here, we extended cLBM [9] to 3D systems. The system is discretized in cubic boxes of side Δ*x* (mm). At every time step Δ*t_CN_,* an amount *F_d,met_* of metabolite *met* (in mmols per box) will move from box (*i,j,k*) to one of the 6 neighboring boxes, indicated as (*i+1,j,k*) for *d*= 1, (*i-1,j,k*) for *d*= 2, (*i,j+1,k*) for *d*= 3, (*i,j-1,k*) for *d*= 4, (*i,j,k+1*) for *d*= 5, (*i,j,k-1*) for *d*= 6, only if there is enough free space in the target box. This is determined by the probability *P_met_*, such that [9]:

$F_{d,met}\left( {i,j,k}_{next},t+\Delta t \right)=\left( F_{d,met}\left( {i,j,k}_{next},t \right)+\Omega_{d,met}^{diff}\left( i,j,k,t \right) \right)P_{met}\left( {i,j,k}_{next},t \right). d=1,\ldots,6$ (S13a)

$F_{0,met}\left( i,j,k,t+\Delta t \right)=\rho_{met}\left( i,j,k,t \right)-\sum_{d} F_{d,met}\left( {i,j,k}_{next},t+\Delta t \right).$ (S13b)

*F_0,met_* represents the amount of metabolite *met* that remains in the same box (*i,j,k*). As in the CN approach, *P_met_* = *γ_met_^-1^* is computed by SPT (Eq. 5 of the main text).

Based on the Bhatnagar-Gross-Krook (BGK) approximation, the diffusion collision term $\Omega_{d,met}^{diff}$ of Eq. S13a can be written as [11]

$\Omega_{d,met}^{diff}\left( i,j,k,t \right)=\omega_{met}\left( F_{d,met}^{eq}\left( i,j,k,t \right)-F_{d,met}(i,j,k,t) \right)$. (S14)

The equilibrium distribution function $F_{d,met}^{eq}$ is given by [9]

$F_{d,met}^{eq}\left( i,j,k,t \right)=\frac{1}{2dim}\rho_{met}\left( i,j,k,t \right).$ (S15)

Where the factor 1/2dim is associated with the number of neighboring boxes (dim = 2 for 2D systems and dim = 3 for 3D), while the macroscopic density of metabolites *met*, *ρ_met_* (in mmol per box), is estimated as

$\rho_{met}\left( i,j,k,t \right)=\sum_{d} F_{d,met}(i,j,k,t)$. (S16)

The relationship between the size of the boxes Δ*x* and the time step Δ*t* is given by the relaxation parameter *ω_met_* (dimensionless), so that

$\omega_{met}=\frac{2}{1+6D_{met}^{o}\frac{\Delta t}{{\Delta x}^{2}}},$ (S15)

where $D_{met}^{o}$ (mm^2^ s^-1^) is the diffusion coefficient in water. In cLBM, the selection of the parameters Δ*x* and Δ*t* must satisfy the constraint *ω_met_* ≤ 1 in order to avoid numerical problems. The most accurate diffusion solution is found when *ω_met_* = 1 [9].

Unlike the semi-implicit CN approach that is stable for large Δ*t_CN_*, cLBM is formulated as an explicit method where the maximum time step is limited to $\Delta t_{CN}\leq\frac{\Delta x^{2}}{6D_{met}^{o}}$ [12]. Nevertheless, cLBM allows the computation of the mean squared displacement (MSD) of the molecules, which is useful for studies of anomalous diffusion [13]. MSD of *met* molecules across the system can be estimated as

${MSD}_{met}\left( t+\Delta t \right)=\frac{\sum_{ijk}^{N_{voxels}} \sum_{d=1}^{6} F_{d,met}\left( ijk,t+\Delta t \right)N_{A}\Delta x^{2}}{\sum_{ijk}^{N_{voxels}} \sum_{d=0}^{6} F_{d,met}\left( ijk,t+\Delta t \right)N_{A}}.$ (S16)

The denominator in Eq. S16 represents the total number of molecules of *met* in the system at time *t* + Δ*t*. *N_A_* is Avogadro number (in molecules per mmol).

# **IbM rules**

The behavior of microbial cells is governed by a series of rules. These IbM rules (described below) are applied individually to each agent (or cell) in the system at every Δ*t*.

*Cell properties.* As a result of the metabolic activity, cell division, motion of the cells, etc., some cell properties such as the mass and radius (Eq. 6 and 7 of the main text, respectively) change during the IbM simulations, which eventually affect the cellular processes and metabolite diffusion. Thus, cell properties such as box position *ijk*, phenotype *Phen*, growth rate *v_bio_*, mass *M_cell_*, and radius *R_cell_* are tracked at every time step and are used to update the local crowding conditions and the composition of the microbial community.

*Cell division.* If *M_cell_* at time *t* reaches *M_max,sp_* (the maximum dry mass before the cell replication take place), then the cell will divide into two identical cells of mass 0.5*M_max,sp_*. Asymmetric cell division can also be applied in CROMICS simulations. The daughter cell is allocated in a randomly selected empty neighboring box, while the mother cell remains in the current site *ijk*. Each IbM-box containing a cell is surrounded by *n_shov_* boxes (Moore neighborhood was chosen to streamline the search for empty boxes to allocate the daughter cell), where *n_shov_* is equal to 8 for 2D systems and 18 for 3D systems. When all neighboring boxes are occupied by cell agents, the daughter cell will randomly choose one of the *n_shov_* directions with the nearest empty box, and push/displace all cells located in the direction line one box away. The empty box created next to the mother cell will be then occupied by the daughter cell. This process is known as shoving.

*Cell death.* Alternatively, if the cell in box *ijk* has been under starvation conditions so that *M_cell_* is less than the minimal dry mass threshold *M_min,sp_*, then the cell dies and is removed from the system.

*Cell motion.* Additionally, if cell division or death is not carried out at time *t*, i.e., *M_min,sp_* ≤ *M_cell_*(*ijk*,*t*+Δ*t*) ≤ *M_max,sp_*, a cell growing in a planktonic state (i.e., swimming in a liquid medium) can move around the system. Two types of *cell motion* can be distinguished, non-motile cells describe a random walk (or Brownian motion), while motile cells can sense the levels of certain substances and perform a biased walk towards an attractant (e.g. substrates) or away from a repellent (e.g. poison).

The random/biased walk of a cell *x* initially located in box *ijk* is simulated using Monte Carlo algorithm rules [14]. Here the cell randomly chooses a neighboring box *ijk_next_* based on the weights *w_vox_* assigned to each *n_vox_* neighboring sites (Von Neumann neighborhood). For a random walk, *w_vox_* is equal to 1/*n_vox_*, where *n_vox_* is equal to 4 for 2D systems and 6 for 3D systems. For a biased walk (chemotaxis), *w_vox_*(*ijk_next_*) can be a function of the concentration gradient of metabolite *met* between the boxes *ijk_next_* and *ijk*, where a box *ijk_next_* is more likely to be chosen if *w_vox_*(*ijk_next_*) is greater than the weights of the other neighbors. If box *ijk_next_* is free, then the cell will move to this with a probability *P_sp_* defined as [15]

$P_{sp}=n_{vox}D_{sp}\Delta t/\Delta x^{2}$, (S17)

where *D_sp_* is the diffusion coefficient of species *sp*. If box *ijk_next_* is already occupied by other agents, then the cell will remain in the original position *ijk*. Thus, the non-overlapping restriction is satisfied in a straightforward manner.

**SI References**

1. Minton AP: The influence of macromolecular crowding and macromolecular confinement on biochemical reactins in physiological media. *J Biol Chem.* 2001; 20:2093–2120.
2. Angeles-Martinez L, Theodoropoulos C: The influence of crowding conditions on the thermodynamic feasibility of metabolic pathways. *Biophys J.* 2015; 109:2394–2405.
3. Park JM, Kim TY, and Lee SY: Constraints-based genome scale metabolic simulation for systems metabolic engineering. *Biotechnol Adv.* 2009; 27:979–988.
4. Henry CS, Broadbelt LJ, Hatzimanikatis V: Thermodynamics-based metabolic flux analysis. *Biophys J.* 2007; 92:1792–1805.
5. [Jankowski MD](https://www.ncbi.nlm.nih.gov/pubmed/?term=Jankowski%20MD%5BAuthor%5D&cauthor=true&cauthor_uid=18645197), [Henry CS](https://www.ncbi.nlm.nih.gov/pubmed/?term=Henry%20CS%5BAuthor%5D&cauthor=true&cauthor_uid=18645197), [Broadbelt LJ](https://www.ncbi.nlm.nih.gov/pubmed/?term=Broadbelt%20LJ%5BAuthor%5D&cauthor=true&cauthor_uid=18645197), [Hatzimanikatis V](https://www.ncbi.nlm.nih.gov/pubmed/?term=Hatzimanikatis%20V%5BAuthor%5D&cauthor=true&cauthor_uid=18645197): Group contribution method for thermodynamic analysis of complex metabolic networks. [*Biophys J*](https://www.ncbi.nlm.nih.gov/pubmed/18645197)*.* 2008; 95:1487–99.
6. Jain AK, Mao J, Mohiuddin KM: Artificial neural networks: A tutorial. *Computer.* 1996; 29:31–44.
7. Cen W, Hoppe R, Gu N. Fast and accurate determination of 3D temperature distribution using fraction-step semi-implicit method. *AIP Advances*. 2016; 6: 095305.
8. Yanenko NN. The Method of Fractional Steps. Berlin, Heidelberg, New York: Springer; 1971.
9. Angeles-Martinez L, Theodoropoulos C: A lattice Boltzmann scheme for the simulation of diffusion in intracellular crowded systems. *BMC Bioinformatics.* 2015; 16:353.
10. Muramatsu N, Minton A. Tracer diffusion of globular proteins in concentrated protein solutions. *Proc Natl Acad Sci USA.* 1988; 85:2984–8.
11. McNamara GR, Zanetti G. Use of the Boltzmann equation to simulate lattice-gas automata. *Phys Rev Lett.* 1988; 61:2332–5.
12. Wolf-Gladrow DA: Lattice-Gas Cellular Automata and Lattice Boltzmann Models – An Introduction. Berlin, Heidelberg, New York: Springer; 2005.
13. Vilaseca E, Isvoran A, Madurga S, Pastor I, Garcés JL, Mas F: New insights into diffusion in 3D crowded media by Monte Carlo simulations: effect of size, mobility and spatial distribution of obstacles. *Phys Chem Chem Phys.* 2011; 13*:*7396.
14. Berry H: Monte Carlo simulations of enzyme reactions in two dimensions: fractal kinetics and spatial segregation. *Biophys J.* 2002; 83:1891–1901.
15. Ridgway D, Broderick G, Lopez-Campistrous A, Ru’aini M, Winter P, Hamilton M, Boulanger P, Kovalenko A, Ellison MJ: Coarse-grained molecular simulation of diffusion and reaction kinetics in crowded virtual cytoplasm. *Biophys J.* 2008; 95:3748–3759.
